# mRNA vaccines transform personalized lung cancer treatment

**DOI:** 10.3389/fimmu.2025.1707654

**Published:** 2026-02-12

**Authors:** Kaiqi Wei, Zitong Wan, Miaomiao Wen, Shaowei Xin, Yinxi Zhou, Jun Wei, Jianfei Zhu, Chengbin Tang, Yanlu Xiong, Tao Jiang, Jie Lei

**Affiliations:** 1 Department of Thoracic Surgery, Tangdu Hospital, Fourth Military Medical University, Xi’an, China; 2 College of Life Sciences, Northwestern University, Xi’an, China; 3 Department of Thoracic Surgery, Air Force Medical Center, Fourth Military Medical University, Beijing, China; 4 Department of Thoracic Surgery, 962 Hospital of the joint Logistics Support Force, Harbin, China; 5 Department of Thoracic Surgery, Air Force 986 Hospital, Xi’an, China; 6 Department of Thoracic Surgery, Shaanxi Provincial People’s Hospital, Xi’an, China; 7 Department of Thoracic Surgery, First Medical Center, Chinese People‘s Liberation Army (PLA) General Hospital and People‘s Liberation Army (PLA) Medical School, Beijing, China; 8 Innovation Center for Advanced Medicine, Tangdu Hospital, Fourth Military Medical University, Xi’an, China

**Keywords:** cancer vaccines, immune subtypes lung cancer immunotherapy, lung cancer, mRNA vaccines, precision treatment, tumor antigens

## Abstract

Targeted therapy and immunotherapy represent major innovations in the treatment of lung cancer. However, in patients with driver gene-positive tumors, the emergence of acquired resistance to targeted drugs is inevitable. As for immunotherapy, its efficacy in early-stage lung cancer patients remains uncertain due to strong immune heterogeneity. In advanced and locally advanced patients, high tumor mutational burden leads to significant genomic instability and tumor progression, and resistance still inevitably develops even with standard chemotherapy combined with immunotherapy. Cancer vaccines, as an approach that activates the antitumor immune cycle from its origin, offer advantages such as the ability to target multiple antigens, minimal off-target effects, a wide therapeutic window, and low toxicity. Furthermore, such vaccines can induce long-lasting immune memory and possess a certain capacity to remodel the tumor immune microenvironment, which helps prevent cancer recurrence, demonstrating broad prospects in lung cancer treatment. Currently, various types of tumor vaccines (including those based on microorganisms, peptides, proteins, exosomes, and DNA) have been developed, yet they often face limitations in safety, insufficient personalization, and immature production pipelines. In contrast, messenger RNA (mRNA)-based vaccines offer distinct advantages, including the efficient generation of protective immune responses, relatively low side effects, and lower acquisition costs, making them a forefront option for novel lung cancer therapies. This review summarizes the current research status of lung cancer vaccines, clarifies the unique therapeutic advantages of mRNA vaccines compared to traditional vaccine modalities, and highlights existing challenges associated with mRNA vaccines. It also provides an overview of current clinical trials of mRNA vaccines for lung cancer and proposes rational design and clinical application strategies for personalized mRNA vaccines within the framework of precision oncology, based on evidence-based medicine.

## 1 Introduction

Lung cancer remains the leading cause of cancer-related mortality worldwide, with non-small cell lung cancer (NSCLC) accounting for over 80% of all cases (1–3). Factors such as histological subtype, patient age, tumor location, disease stage, drug tolerance, and patient preference influence the first-line treatment choices, which include surgery, radiation, chemotherapy, targeted therapy, immunotherapy, and combination therapies.

**Figure 1:**
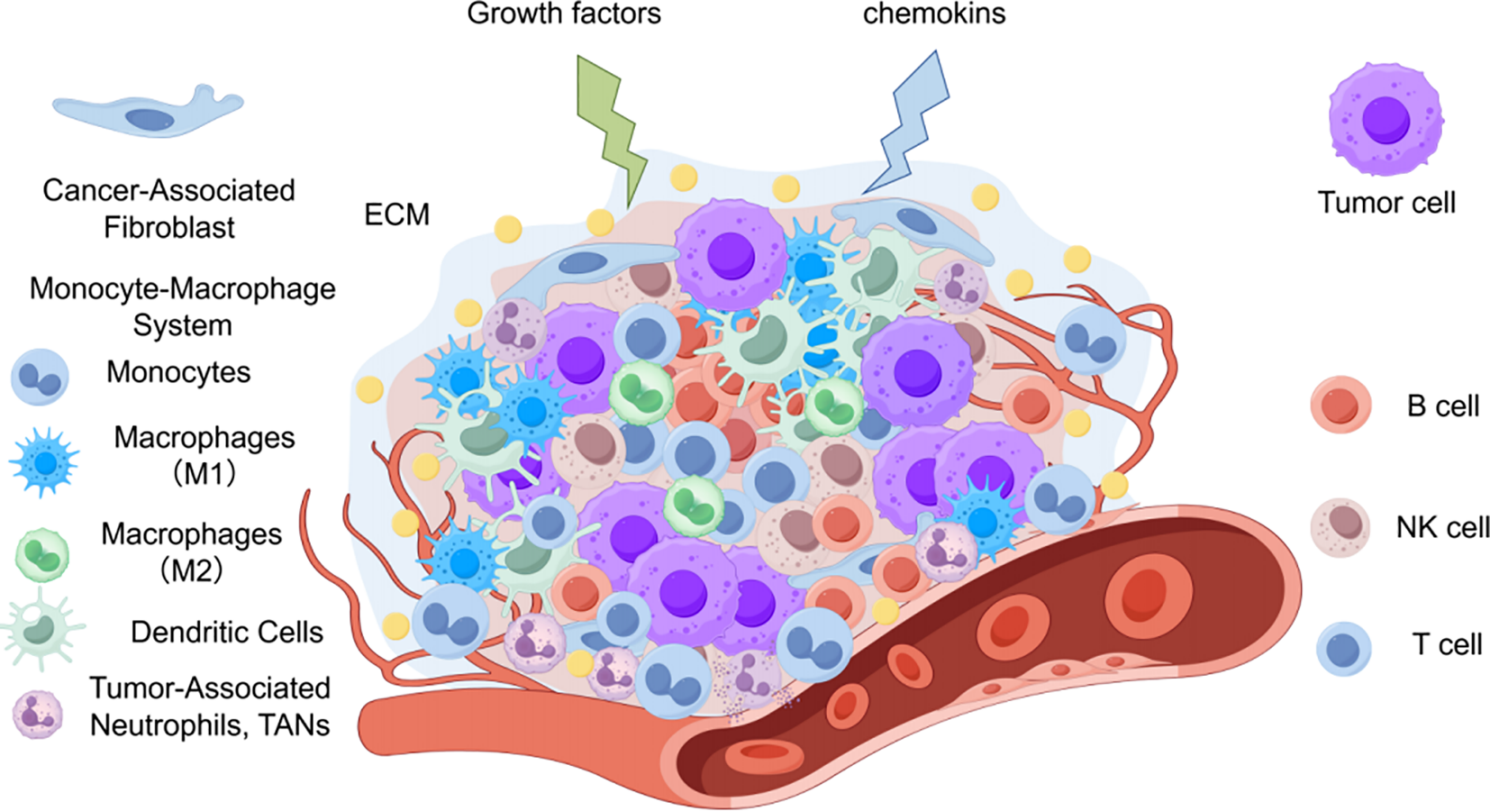
The tumor microenvironment (TME) further complicates treatment outcomes through dynamic crosstalk among stromal components, immune infiltrates, and extracellular matrix constituents, creating barriers to immune cell infiltration and function.

Although the application of targeted therapies and immune checkpoint inhibitors (ICIs) has significantly improved the prognosis for patients with advanced NSCLC, core challenges such as tumor heterogeneity, acquired resistance, and immunosuppressive microenvironments continue to limit further gains in therapeutic efficacy (4). (**Figure 1**) For example, tyrosine kinase inhibitors (TKIs) targeting driver genes like EGFR and ALK, while initially effective, ultimately lead to resistance in most patients and is not applicable for negative driven-gene patients (5–7).

Over the past decade, a paradigm shift has occurred with the emergence of immune-based interventions as transformative therapeutic modalities, centered on the strategic manipulation of immune recognition and effector mechanisms to reinvigorate host antitumor immunity, particularly by restoring cytotoxic T lymphocyte (CTL) activity against tumor antigens (8, 9). However, the efficacy of these approaches is frequently subverted by tumor-intrinsic adaptations—such as antigenic drift and immune checkpoint upregulation—and the immunosuppressive tumor microenvironment (10, 11) (**Figure 1**). Consequently, immunotherapy often shows limited or unsustainable responses in a subset of patients and can lead to clinically significant adverse events such as checkpoint inhibitor pneumonitis (CIP), which may necessitate treatment discontinuation (12). Furthermore, even initially responsive patients frequently develop acquired resistance after a period of treatment. In this context, therapeutic cancer vaccines represent a promising strategy to overcome these limitations. For instance, in a randomized open-label study in solid tumors, the cancer vaccine OSE2101 demonstrated significant advantages over chemotherapy: its objective response rate (ORR) reached 33.3%, compared to 18.2% in the chemotherapy group, and median progression-free survival (mPFS) was extended to 6.8 months versus 4.3 months, alongside a more favorable safety profile. These results suggest that such vaccines can effectively counteract immunotherapy resistance and improve patient outcomes. Supporting this, another double-blind controlled trial showed that a vaccine-based combination therapy increased median overall survival (mOS) in advanced solid tumor patients to 15.6 months, significantly longer than the 10.2 months with chemotherapy alone, while also exhibiting a lower incidence of treatment-related adverse events, highlighting its potential to enhance survival quality (13). As a major focus in contemporary oncology, mRNA vaccines may therefore reshape the current therapeutic landscape for lung cancer.

In recent years, messenger RNA (mRNA)-based therapeutic vaccines have emerged as a frontier in lung cancer immunotherapy. The fundamental impetus for their development lies in leveraging the high tumor mutational burden (TMB) characteristic of lung cancer (14). The abundance of mutations generates a rich repertoire of tumor neoantigens, providing an ideal target library for vaccines (15). By encoding these neoantigens, mRNA vaccines guide the host immune system to specifically recognize and eliminate tumor cells. This approach not only enables highly personalized treatment but also fosters long-term anti-tumor effects through the induction of T-cell immune memory. Furthermore, combining mRNA vaccines with immune modulators holds the potential to more effectively reverse the tumor-suppressive microenvironment (16).

From a technological perspective, mRNA vaccines offer distinct advantages in their high programmability and rapid manufacturing capability. Advances in lipid nanoparticle (LNP) delivery systems have addressed historical challenges related to mRNA stability and *in vivo* delivery efficiency (17). Moreover, the integration of artificial intelligence and multi-omics data for antigen selection has made the rapid design of “personalized neoantigen vaccines” tailored to a patient’s unique mutational profile a tangible possibility (18).

Currently, several lung cancer mRNA vaccine candidates have progressed to the clinical validation stage. For instance, the BNT116 vaccine has demonstrated favorable safety and preliminary anti-tumor activity in an early-phase clinical trial (NCT05142189) (19). More innovatively, LungVax is poised to become the world’s first experimental preventive vaccine for lung cancer, with clinical trials scheduled to commence in 2026. It aims to intercept cancer development at its origin by identifying and clearing precancerous lesions. Concurrently, researchers in China are actively exploring this field; for example, a team from West China Hospital has developed a personalized dendritic cell (DC) vaccine successfully applied in post-operative lung cancer patients, demonstrating the feasibility of precision immunotherapy (20).

Therefore, this review aims to provide an in-depth exploration of the current landscape of mRNA vaccine development for lung cancer. We will focus on analyzing how these vaccines leverage the genetic mutation profiles of lung cancer to overcome existing therapeutic barriers, review key technological advances and challenges, analyze the specific underlying issues, and discuss the clinical translation prospects of mRNA vaccines as a next-generation modality for precision immunotherapy.

## 2 mRNA vaccines

### 2.1 Historical context of mRNA vaccines

**Figure 2:**
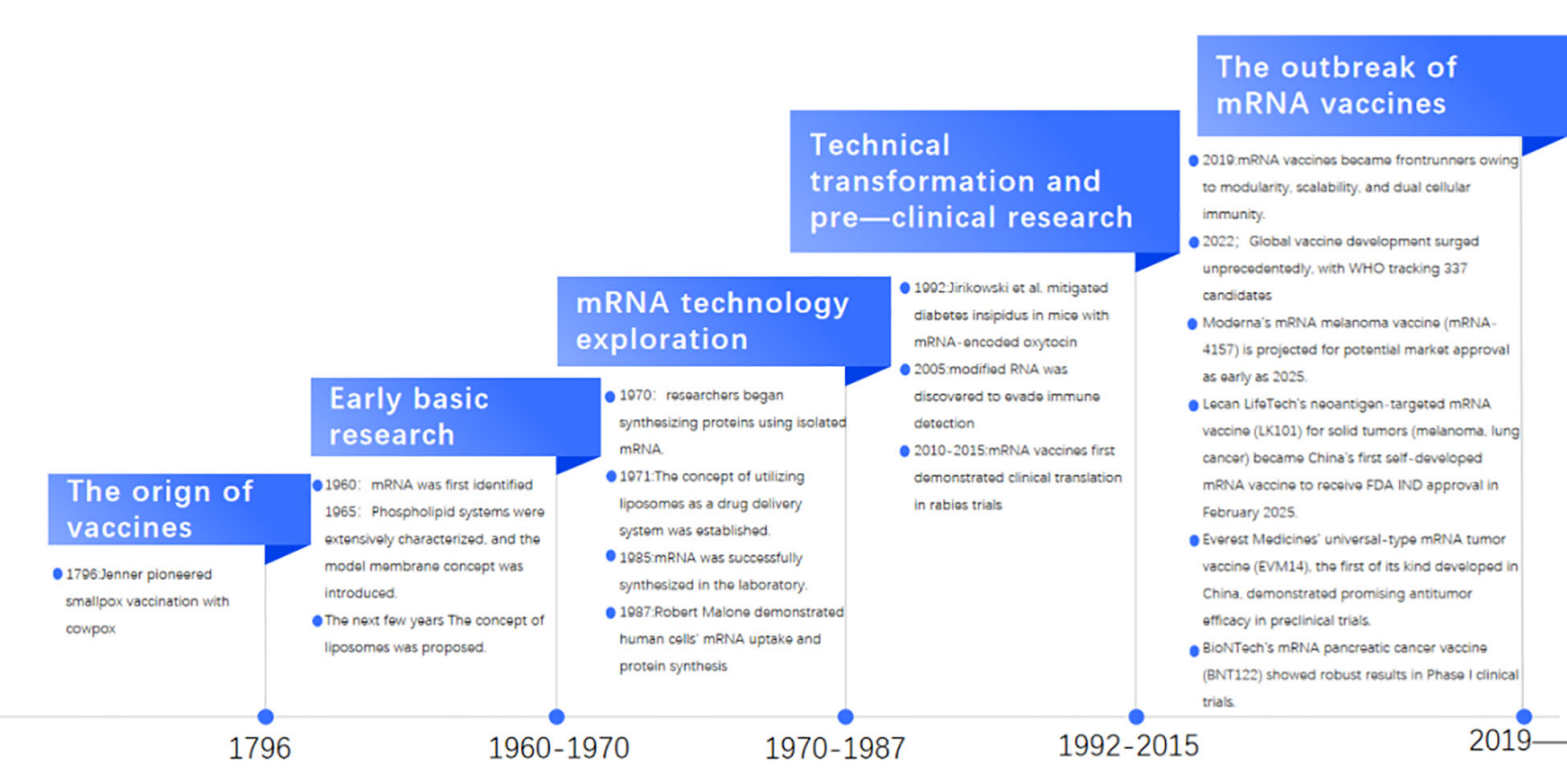
Historical context of mRNA vaccines.

mRNA vaccines have recently emerged as an effective tool for preventing and treating various diseases. The use of vaccines in medicine has a long history, dating back to 1796 when Edward Jenner successfully vaccinated a boy against smallpox using the cowpox vaccine (21) (**Figure 2**). However, the discovery and application of mRNA are relatively recent, with only a few decades of history. mRNA was first identified in 1960; in the 1970s, researchers began synthesizing proteins using isolated mRNA; by 1985, mRNA was successfully synthesized in the laboratory.

A pivotal milestone in the development of mRNA vaccines occurred at the end of 1987 when Robert Malone discovered that human cells could absorb mRNA and produce the corresponding proteins following exposure to a mixture of mRNA strands and lipid droplets (22). This established a foundation for the subsequent research and development of mRNA vaccines. Research into this area has gradually increased over the years. In 1992, Jirikowski et al. successfully alleviated symptoms of diabetes insipidus in a murine model by administering mRNAs encoding oxytocin and vasopressin (23). The authors demonstrated the potential of mRNA in disease treatment. In the 21^st^ century, research on mRNA vaccines has made significant breakthroughs. In 2005, modified RNA was discovered to evade immune detection, providing novel insights for mRNA vaccine design. The translational potential of mRNA vaccine platforms was first demonstrated in clinical trials targeting rabies virus between 2010 and 2015, establishing foundational safety and immunogenicity profiles (24, 25). The unprecedented challenges posed by the COVID-19 pandemic catalyzed rapid technological advancements, with mRNA vaccines emerging as frontrunner candidates due to their modular design architecture, scalable production pipelines, and potent humoral/cellular immune induction (26). Global vaccine development efforts reached an unprecedented scale, with WHO tracking 337 candidate vaccines by 2022, including 47 mRNA-based formulations, of which 23 progressed to clinical evaluation (26). This accelerated trajectory validated mRNA platforms’ pandemic responsiveness and redefined vaccine development paradigms, positioning nucleic acid-based approaches as essential tools for addressing emerging infectious threats. BioNTech, Moderna, and CureVac, leveraging their profound technological expertise, constitute the core forces in the current pipeline landscape. These pipelines primarily follow two strategies: personalized neoantigen vaccines based on patient-specific mutations and “off-the-shelf” vaccines targeting shared tumor-associated antigens.

BioNTech has established the most extensive mRNA cancer vaccine pipeline, adopting a dual-track development strategy of both “personalized” and “off-the-shelf” approaches. Fixed-Antigen Vaccine BNT116 for Advanced NSCLC: BNT116 is a vaccine containing mRNAs encoding six common tumor-associated antigens in NSCLC (including CLDN6, PRAME, etc.). Its Phase I clinical data presented at the 2025 American Association for Cancer Research (AACR) Annual Meeting were encouraging. The study showed that BNT116 combined with the PD-1 inhibitor cemiplimab, used as first-line treatment for advanced NSCLC patients unsuitable for platinum-based chemotherapy, achieved an 80% disease control rate, a 45% objective response rate, and induced rapid ctDNA clearance. This indicates that mRNA vaccines can effectively break tumor immune tolerance and synergize with immune checkpoint inhibitors (27). Synergistic Layout of Personalized Neoantigen and Fixed-Antigen Vaccines: In addition to BNT116, its personalized neoantigen vaccine BNT122 (in collaboration with Roche) is advancing in late-stage clinical trials across multiple cancer types. Concurrently, the success of its fixed-antigen vaccine BNT111 in Phase II trials for melanoma further validates the breadth of its platform technology. The company also plans to initiate a global clinical trial in 2025 combining BNT327/PM8002 (a bispecific antibody) with an mRNA cancer vaccine, aiming to establish a next-generation backbone for lung cancer immunotherapy.

The collaboration between Moderna and Merck is a benchmark in the field of personalized mRNA cancer vaccines, with its core product, mRNA-4157 (V940), achieving breakthrough results in clinical research. Breakthrough Efficacy of mRNA-4157: This vaccine identifies patient-specific tumor mutations through sequencing and synthesizes mRNA encoding up to 34 neoantigens. In a Phase III clinical trial for post-surgical adjuvant treatment of high-risk melanoma, combining mRNA-4157 with pembrolizumab (Keytruda) alone significantly reduced the risk of recurrence or death compared to pembrolizumab monotherapy. Based on this success, the partners have initiated a Phase III study in non-small cell lung cancer (NSCLC) to validate this strategy in lung cancer (28). Pipeline Expansion and Technological Iteration: Moderna’s other candidate, mRNA-4359, is designed to target the immunomodulatory enzyme indoleamine 2,3-dioxygenase 1 (IDO1), tumor-associated antigens (TAAs), and the immune checkpoint molecule programmed death-ligand 1 (PD-L1), aiming to overcome the immunosuppressive tumor microenvironment and demonstrating potential immunostimulatory and anti-tumor activity. It has shown promise in early-stage clinical trials for patients resistant to immunotherapy. Furthermore, Moderna has established a highly automated, dedicated manufacturing facility for its personalized cancer vaccine mRNA-4157, laying the groundwork for addressing the production bottlenecks of personalized vaccines (29).

Following an adjustment in its infectious disease vaccine strategy, CureVac is shifting its focus to oncology and attracting significant attention due to its integration with BioNTech. Next-Generation Lung Cancer Vaccine CVHNLC: Its “off-the-shelf” candidate vaccine CVHNLC for squamous non-small cell lung cancer (sqNSCLC) received clinical trial approval from the European Medicines Agency (EMA) in 2025, marking its official entry into clinical development for lung cancer treatment. Merger Synergy with BioNTech: In 2025, BioNTech announced the acquisition of CureVac. This move aims to integrate their respective expertise in mRNA design, delivery technologies, and manufacturing to accelerate the development of mRNA cancer immunotherapies. This industry consolidation is expected to significantly enhance BioNTech’s R&D capabilities in key areas such as lung cancer. Beyond pipeline expansion, mRNA technology itself is continuously evolving. A study published in August 2025 proposed a “minimalist mRNA” design strategy. The research posited that for small antigenic molecules expressed by antigen-presenting cells, the untranslated regions (UTRs) and non-coding regions traditionally considered essential in conventional mRNAs might be dispensable. This simplified mRNA not only maintains effective immunogenicity but also accelerates candidate antigen screening and vaccine production processes, offering a new technological pathway for the rapid manufacturing of personalized cancer vaccines (30).

### 2.2 Types and development of mRNA vaccines

As key intermediate products from transcription to translation, mRNA carries the genetic code that guides the synthesis of the corresponding proteins. mRNA vaccines, a significant subset of nucleic acid vaccines, can be categorized into two primary types based on their characteristics: self-amplifying RNA (saRNA) and non-replicating mRNA. saRNA viral vaccines leverage the efficient amplification characteristics of single-stranded RNA within host cells to generate vaccines containing genes for viral replication and therapeutic purposes. Based on the methods used to acquire antigen expression, saRNA vaccines can be classified into three types: those derived from DNA plasmids, those delivered via virus-like particles, and those utilizing *in vitro* transcribed and amplified RNA. Immunological studies conducted in animal models have demonstrated that saRNA vaccines elicit robust cellular and humoral immune responses (31). Traditional non-replicating mRNA vaccines possess a relatively straightforward architecture, comprising a cap structure, a 5′-untranslated region (UTR), an open reading frame (ORF) encoding the vaccine antigen, a 3′-UTR, and a poly(A) tail. In addition to the ORF, which directly encodes the antigen, the other structural components are essential for maintaining mRNA stability and augmenting transcriptional efficiency. These elements also serve as modification sites that can prolong the *in vivo* half-life of mRNA and effectively mitigate unwanted immune responses (32). Compared with saRNA, conventional non-replicating mRNA vaccines are distinguished by their compact size and relatively simple structure, which includes a single ORF for encoding vaccine antigens. This design strategy ensures the vaccine elicits an immune response against specific antigens, avoiding unnecessary immune responses (33).

This fundamental mechanistic difference directly leads to distinct characteristics in antigen expression and immune responses between the two. Non-replicating mRNA enables rapid initiation of high-level antigen expression, but its duration is relatively short, typically lasting several days to a week. This “high-intensity, short-pulse” pattern effectively activates both cellular immunity (CD8^+^ T cells) and humoral immunity. In contrast, while saRNA may have a lower initial antigen expression level, its self-amplifying capability allows for sustained antigen expression lasting weeks or even months. This “low-dose, long-term” antigen exposure pattern is considered more conducive to inducing strong and long-lasting T cell immunological memory, which is crucial for eliminating tumor cells and preventing cancer recurrence (33). However, advantages coexist with challenges. The larger molecular size of saRNA—typically two to three times that of non-replicating mRNA—places higher demands on its production and delivery. Its complex sequence poses challenges for the design of plasmid DNA (pDNA) templates and the stability of *in vitro* transcription, while also requiring higher efficiency from delivery systems. In contrast, non-replicating mRNA benefits from its smaller molecular weight and simpler structure, resulting in a more mature and stable production process, as well as higher efficiency in encapsulation by delivery systems such as lipid nanoparticles (LNPs) (34).

Overall, these two platforms each have distinct emphases in their application strategies for cancer vaccines. Non-replicating mRNA technology is well-established and capable of rapidly eliciting potent immune attacks, making it highly suitable for developing therapeutic vaccines targeting personalized neoantigens with the aim of quickly eliminating existing tumors. In contrast, saRNA demonstrates unique potential in adjuvant vaccine scenarios that require the establishment of long-term immune surveillance to prevent tumor recurrence, thanks to its persistent antigen expression characteristics. Currently, personalized neoantigen vaccines represented by BioNTech and Moderna predominantly employ the non-replicating mRNA platform and have advanced to late-stage clinical trials. Meanwhile, saRNA vaccines developed by several companies are largely in early-stage clinical exploration for infectious diseases and oncology, with their clinical potential awaiting further validation.

Over the past few decades, the development of mRNA vaccines has encountered several significant challenges, including the intrinsic instability of mRNA molecules, their strong immunogenicity, and the lack of effective mRNA delivery systems (11). With continuous advancements in science and technology, these difficulties are gradually being resolved. First, mRNA vaccines offer unique advantages, as they do not use replicating vectors and hence lack issues such as antibiotic resistance, genomic integration risks, or strong immunogenic reactions (35). This characteristic has enabled mRNA vaccines to safely induce antibody responses in phase I clinical trials (21).

Besides tackling the issues of mRNA instability and immunogenicity, modified mRNA can remarkably minimize adverse reactions and enhance therapeutic effectiveness. Current investigations are delving into diverse mRNA delivery approaches. These encompass lipid-based, polymer-based, and peptide-based delivery platforms, along with cationic nanoemulsions. These delivery systems not only improve the stability of mRNA but also enhance its cellular uptake and penetration, making mRNA vaccines more effective in overcoming the initial challenges (36). Notwithstanding the substantial headway in advancing mRNA vaccines, this domain remains in an early and evolving phase. Consequently, it is essential to remain alert and prioritize safety-related factors when furthering the mRNA vaccine. Nevertheless, with ongoing advancements and technological improvements, the future of mRNA vaccines appears promising.

## 3 Lung cancer vaccines

### 3.1 Conventional lung cancer vaccines

**Figure 3:**
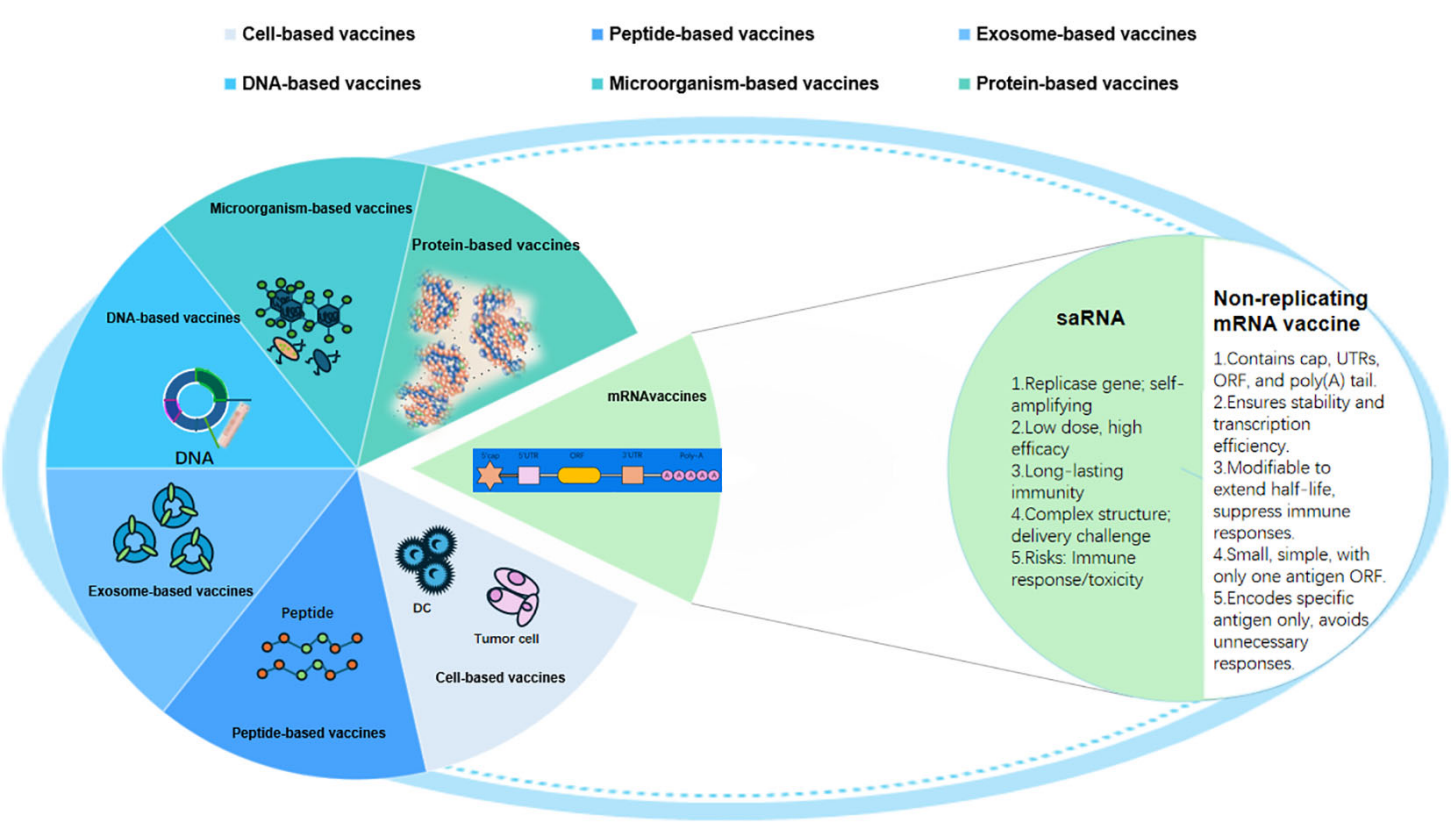
Classification of existing lung cancer vaccines. Multiple lung cancer vaccines have been developed to date, including cell−based, microorganism−based, exosome−based, protein−based, peptide−based, DNA−based, and mRNA vaccines. DC, dendritic cell.

Currently, various traditional vaccines are being developed for lung cancer treatment. These vaccines can be broadly categorized into antigen-specific vaccines, which include peptide/protein, DNA, and vector-based vaccines, and whole-cell vaccines, such as allogeneic and autologous DC vaccines (**Figure 3**).

#### 3.1.1 Peptide/protein vaccines

The protein-specific vaccines currently employed for NSCLC treatment include CIMAvax-EGF, which targets the epidermal growth factor (EGF); melanoma-associated antigen A3 (MAGE-A3); New York esophageal squamous cell carcinoma (NY-ESO-1); the BLP25 liposome vaccine, which targets mucin 1 (MUC1). Montanide ISA 51, a partial enhancer, is utilized to elicit a targeted immune response against EGF (37). In phase II randomized controlled trials and phase III studies involving advanced NSCLC patients, CIMAvax-EGF has exhibited excellent safety profiles and potent immunogenicity, leading to notable improvements in survival outcomes. In phase I/II clinical trial (NCT02955290) where CIMAvax-EGF was combined with the monoclonal antibody nivolumab for the treatment of NSCLC, patients with low expression of programmed death-ligand 1 (PD-L1) in their tumors, who had a suboptimal response to nivolumab monotherapy, demonstrated promising therapeutic effects upon receiving the combined treatment of the vaccine and immunotherapy. Biomarker analysis indicated that this combination elicits a stronger immune response than CIMAvax, which was administered alone to patients (38). Current antitumor therapy places greater emphasis on activating specific cellular immune responses. However, the primary mechanism of action of this vaccine is to activate humoral immunity. It remains unknown whether this is the reason why the vaccine’s efficacy has not been further improved. Additionally, not all patients benefit equally from this vaccine. A biomarker—baseline EGF levels—is required to identify the population most likely to benefit. Furthermore, Phase III trials have indicated that patients need to complete an initial induction phase of at least four doses of the vaccine to observe a significant survival advantage. This places demands on patient treatment compliance and the follow-up management capabilities of the healthcare system. In the current era of immunotherapy, whether CIMAvax-EGF should be used as maintenance therapy for specific populations or combined with other drugs (such as immune checkpoint inhibitors) to enhance efficacy remains to be clarified and requires more clinical data to define the optimal strategy (39). Cancer-testis antigens are among the proteins targeted by tumor vaccines. In NSCLC, NY-ESO-1 and MAGE-A3 are two representatives of such antigens. Nevertheless, in the phase III MAGRIT study, when patients were administered MAGE-A3, no improvement in disease-free survival was observed compared to those in the dummy treatment control group (40). Besides other antigens, the glycoprotein MUC-1 is expressed in NSCLC tumors, promoting tumor cell proliferation through cell surface receptor interactions (41). The lipopeptide-based vaccine Tecemotide (L-BLP25) was developed as a potential therapeutic agent. In the first-phase clinical investigations, L-BLP25 exhibited high immunogenicity and good tolerance; however, the phase III START trial showed no marked variation in overall survival rates among the group treated with L-BLP25 and the placebo-controlled group (42). The lack of significant improvement in efficacy may be attributed to the rapid disease progression in patients with locally advanced and advanced stages, which may not allow sufficient time for the vaccine to induce a robust immune response capable of activating the immune system to eliminate tumors. Additionally, due to the highly heterogeneous and immunosuppressive tumor microenvironment in locally advanced and advanced non-small cell lung cancer, the induced immune response may be inadequate to effectively overcome such inhibitory mechanisms within the tumor microenvironment (43).

These findings suggest that the research protocol for personalized peptide vaccines in lung cancer treatment requires optimization, and their effects on the immune response and improvement in patient survival remain to be observed.

#### 3.1.2 Vector vaccines

Vector-based vaccines leverage engineered vectors such as bacteria, viruses, or yeasts to express recombinant antigens. TG4010, a vaccine using a viral delivery vehicle, specifically the attenuated vaccinia virus Ankara, contains the coding sequences of human MUC1 and interleukin-2 (IL-2) (44). This vaccine exhibited a favorable safety profile in phase I trials, with only mild local reactions observed. In phase II studies involving patients with advanced or metastatic NSCLC (stages IIIB and IV), combining TG4010 with first-line chemotherapy enhanced antitumor efficacy. A phase II clinical trial evaluating the combination of TG4010 with the immune checkpoint inhibitor nivolumab (NCT00793208) is also underway (45). In the Phase II trial, however, it was observed that approximately one-third of participants experienced adverse reactions following vaccination. Furthermore, viral vector-based vaccines may be limited by the patient’s pre-existing immunity, which can prevent the effective infection of cells and expression of the target antigen, thereby significantly weakening the vaccine’s ability to elicit specific cellular immune responses. Compared to mRNA vaccines, the efficacy of viral vector vaccines is dose-dependent, and higher doses of viral particles are often more likely to trigger strong inflammatory reactions. Additionally, the production of viral vector vaccines relies on mammalian cell culture—a complex, time-consuming, and costly bioprocess. Each vaccine dose requires the cultivation of large quantities of live virus, facing bottlenecks such as lengthy scale-up cycles, high purity requirements, and stringent quality control challenges (46). These findings highlight the promising prospects of vector vaccines for future research. A better understanding of existing vaccines and their molecular mechanisms may improve the therapeutic efficacy of vector-based vaccines.

#### 3.1.3 DC vaccines

As the only cells in the body that can initialize and present antigens, DCs have become a major focus of cellular immunotherapy research. DC vaccines induce antitumor T cell responses and establish immune memory by administering DCs activated by TAAs to patients, thereby preventing tumor recurrence (47). In a first-stage clinical investigation (NCT00601094) including individuals diagnosed with advanced-stage (either IIIB, IV, or recurrent) NSCLC, Lee et al. revealed substantial stimulation of CD8+ T-cell infiltration and antigen-specific immunological reactions. The authors utilized autologous DCs modified with the *CCL21* gene (AdCCL21-DC), highlighting the therapeutic potential of intralesional delivery of autologous DC vaccines targeting lung cancer (48). A Phase I clinical trial (NCT01574222) found the vaccine to be safe, with no evidence of free adenovirus in the peripheral blood post-vaccination and no significant changes in anti-adenovirus antibody levels. Systemic immune responses against tumor-associated antigens (TAAs) were detected via ELISPOT in 6 out of 16 patients. Additionally, the CD8^+^ T-cell infiltration rate increased by an average of 3.4-fold/mm², and 25% of patients (4 out of 16) showed a clinical response of stable disease (SD) at day 56 (49). Recent studies have suggested that the DC vaccine can improve patient survival rates and exhibit great promise when combined with immune checkpoint inhibitor therapy (50, 51). Nevertheless, the inherent biological characteristics and taxonomic categorization of DCs, exacerbated by immunological tolerance, cellular fragility, and restricted viability, hinder their continuous and potent antitumor functionality and present obstacles in manufacturing (51). Therefore, advancing next-generation DC-based immunotherapeutic agents is crucial for surmounting these constraints.

#### 3.1.4 DNA vaccines

DNA-based immunotherapeutic agents employ plasmid vectors that carry the encoding sequences of specific antigens. The employment of this modality confers multiple benefits, such as reusability and cost-effectiveness. Additionally, MHC class I and II molecules present antigens expressed from DNA vaccines, thereby activating CD4+ and CD8+ T cells and inducing antibody-mediated immune responses. Weng et al. demonstrated effective targeting and antitumor responses using a murine lung cancer model generated through genetic engineering vaccinated with a KRas DNA vaccine (43). A MAGE-A3 protein vaccine (recMAGE-A3) was developed to specifically act on the expression of MAGE-A3, which is present in melanoma and NSCLC. Although therapeutic effects were observed in a mouse melanoma model, the large-scale, randomized MAGRIT phase III trial in patients with MAGE-A3-positive NSCLC indicated no significant advantage over placebo, regardless of adjuvant use (52). These studies indicate that although DNA vaccines have shown promising results in animal models, they have failed to achieve similar outcomes in clinical research. It is currently believed that this may be due to antigens such as MAGE-A3 being heterogeneously expressed in tumor cells and potentially lacking high-affinity T cell epitopes, leading to an immune response that cannot cover all tumor cells (escape due to heterogeneity) and struggles to induce potent T cell responses. Additionally, the immune response induced by this vaccine tends to be biased toward specific humoral immunity, which is insufficient to suppress human tumors (45). This may be because animal models such as mice have intact immune systems, the tumor lines used for induction are single and controlled in a uniform environment, which differs significantly from the highly heterogeneous immune status and complex microenvironment of human lung cancer. Vaccine strategies effective in animal models thus fail to reproduce equivalent efficacy in humans. These studies indicate that, although DNA vaccines exhibit good prospects in animal models, they fail to achieve similar results in clinical studies. This highlights the requirement for novel strategies to surmount the obstacles encountered in clinical trials, particularly those based on the high mutation specificity of lung cancer and current prediction algorithms for neoantigen epitopes. The development of DNA vaccines targeting TAAs and TSAs is anticipated to enhance the survival outcomes for individuals afflicted with lung cancer (53).

In summary, conventional vaccines have achieved notable progress in lung cancer treatment. However, given the complexity of preparation and safety concerns associated with these vaccines, a novel type of vaccine to facilitate precise and personalized treatment for lung cancer remains warranted.

## 4 Advantages of mRNA vaccines

As research in immunology and oncology progresses, it is increasingly recognized that somatic mutations in tumor cells can lead to the expression of neoantigens. These neoantigens are recognized by the host immune system and can directly induce tumor cell death (54). Lung cancer is characterized by a high tumor mutational burden, resulting in a rich repertoire of potential neoantigens. These neoantigens, arising from somatic mutations, are key mediators of tumor-specific immune activation and potential targets for personalized lung cancer treatment. The host’s immunological machinery can discern neoantigens, which can directly trigger the apoptosis of malignant cells by activating killer T cells and other immunological effector pathways. Although some neoantigens are difficult to target using other methods, almost all proteins and noncoding RNAs are susceptible to RNA-based therapies (55).

### 4.1 mRNA vaccines trigger innate and adaptive immunity

**Figure 4:**
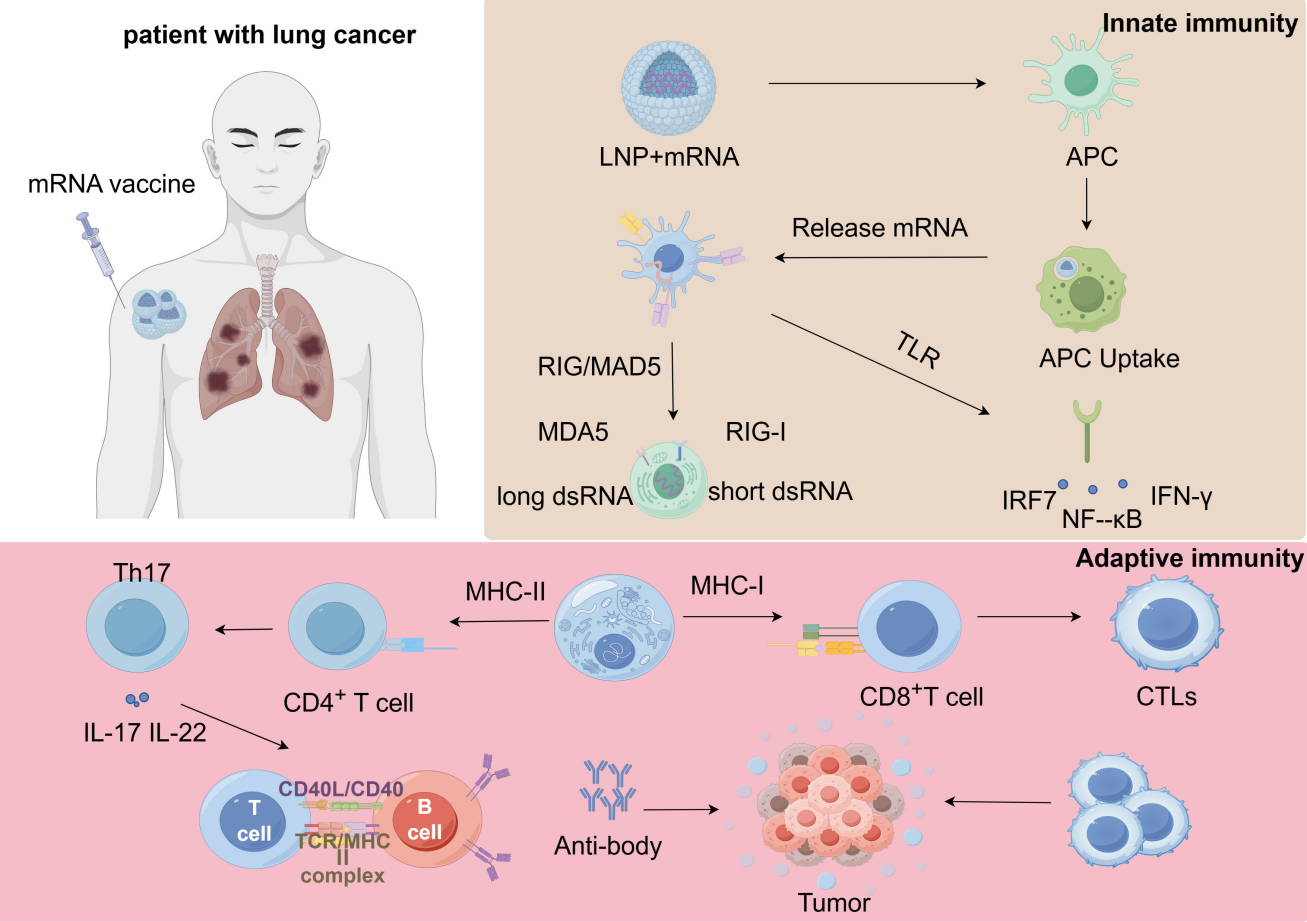
This figure outlines how mRNA vaccines encapsulated in Lipid Nanoparticles (LNPs) activate the immune system. Delivery & Innate Immune Activation: The LNP+mRNA complex is taken up by an Antigen-Presenting Cell (APC). Inside the cell, immune sensors RIG-I and MDA5 recognize mRNA by-products (dsRNA), triggering signaling pathways (IRF7, NF-κB) that launch an innate immune response and release cytokines like IFN-γ.Adaptive Immune Activation: The mRNA is also translated into the target protein (antigen). This antigen is presented on the cell surface by MHC I molecules. The MHC-Antigen complex is recognized by CD8^+^ T cells via their T Cell Receptor (TCR), activating them to become Cytotoxic T Lymphocytes (CTLs). Other immune cells and cytokines (e.g., IL-17, IL-22) contribute to a broader specific immunity. LNP, Lipid Nanoparticle; APC, Antigen-Presenting Cell; RIG-I/MDA5, cytosolic RNA sensors; IRF7/NF-κB, signaling proteins; IFN, Interferon; MHC, Major Histocompatibility Complex; TCR, T Cell Receptor; CTLs, Cytotoxic T Lymphocytes.

mRNA vaccines have the traits of not integrating into the genome and being non-infectious and are accompanied by a transient cellular expression state where repeated administration can be carried out. The encoded sequences in mRNA transcripts show great versatility, allowing for the expression of antigens and molecules that can regulate the immune system to trigger and adjust immune responses of both adaptive immunity and innate immunity. Full-length antigens containing numerous epitopes can be presented to the immune system via MHC-I and MHC-II molecules. Innate immunity serves as the primary defense against nonself antigens (23). Notably, mRNA vaccines primarily activate pro-inflammatory signaling pathways through two sets of pattern-recognition-receptor-mediated mechanisms, which initiate the body’s inborn immune response (56, 57) (**Figure 4**).

This first system is the Toll-like receptor (TLR) system, situated within the plasma membrane, endosomes as well as lysosomes present in epithelial cells and immune cells, such as DCs, monocytes, and macrophages (58). Double-stranded RNA (dsRNA) activates TLR3, which induces the generation of type I interferons (IFNs) through the TRIF (TIR domain-containing adapter-inducing IFN-β) pathway (59). In contrast, single-stranded RNA (ssRNA) activates TLR7 in addition to TLR8 and signals through the MYD88-dependent signaling pathway, resulting in the production of cytokines with pro-inflammatory properties regulated by either nuclear factor (NF)-κB or interferon regulatory factor (IRF) 3. The second system involves the retinoic acid-inducible gene I (RIG-I)-like receptors, being cytosolic RNA sensors playing a vital role in innate antiviral immunity (60, 61).RIG-I and melanoma differentiation-related protein 5 (MDA5) exhibit distinct activation patterns in response to 5′-triphosphorylated dsRNAs of varying lengths: short (18–19 bp) dsRNAs activate RIG-I, while longer (>1,000 bp) dsRNAs activate MDA5 (57, 61). However, excessive activation of these receptors can accelerate vaccine clearance and reduce their protective effects (57, 60). The effectiveness of the activation of the innate immune system instigated by mRNA-based immunotherapies significantly hinges upon the vaccine substances and also upon the manufacturing procedures (62). This encompasses the selection of carriers and the routes of administration, and these aspects represent pivotal areas of current research for mRNA vaccines (63, 64). Following vaccination, antigen-presenting cells (APCs) uptake the mRNA and transport it to the cytoplasm for antigen processing (65). The processed TAAs are then presented on MHC class I and II molecules to activate CD8+ and CD4+ T cells (66, 67). Additionally, CD4+ T cells can co-stimulate antigen-specific B cells, inducing humoral immune responses (68, 69). Conversely, B cells, which also serve in the capacity of APCs, can trigger the activation of CD4+ T cells after engulfing extracellular polypeptides and presenting them on MHC class II molecules, further enhancing the immune response (70). The BNT116 vaccine, currently undergoing clinical trials (NCT05142189), is specifically designed for non-small cell lung cancer (NSCLC). It utilizes lipid nanoparticles (LNPs) to deliver mRNA encoding tumor-associated antigens. After the vaccine is taken up by antigen-presenting cells (such as dendritic cells), the mRNA is translated into complete tumor antigen proteins in the cytoplasm. These antigens are processed and presented via both MHC class I and II molecules, simultaneously activating CD8^+^ cytotoxic T cells and CD4^+^ helper T cells, thereby inducing specific killing of lung cancer cells expressing these antigens. This ability to concurrently stimulate both cellular and humoral immunity represents an advantage of mRNA vaccines over certain traditional platforms (27). KRAS G12V Mutant-Targeting Vaccine: In preclinical studies, an mRNA vaccine specifically designed against the KRAS G12V mutation has demonstrated potential. In lung cancer models, this vaccine elicited robust T-cell responses, notably generating high levels of tumor necrosis factor-alpha (TNF-α) and interferon-gamma (IFN-γ)—cytokines critical for attacking and eliminating tumor cells. This highlights the precision of mRNA vaccines in targeting specific driver gene mutations such as those found in lung cancer (71). mPLA/mRNA Vaccine: A 2023 study reported an mRNA vaccine incorporating monophosphoryl lipid A (mPLA, a TLR4 agonist). In lung cancer models, including advanced models with bone metastasis, this vaccine not only effectively activated T cells but also demonstrated the ability to reprogram the tumor microenvironment: it reversed pro-tumorigenic M2-type macrophages into anti-tumor M1-type macrophages and promoted the infiltration and activation of dendritic cells and natural killer (NK) cells at the tumor site, thereby effectively inhibiting lung cancer growth and metastasis. This example illustrates that through rational design, mRNA vaccines can overcome the immunosuppressive nature of the lung cancer microenvironment (72). Targeted Therapy Precision mRNA functions as a blueprint for polypeptide chain biosynthesis, and the resultant polypeptide chains can undergo post-translational modifications to achieve effective functional conformational folding (23, 49). In addition, mRNA vaccines can produce multimeric polypeptide chains that cannot be correctly folded and assembled during normal biological responses. This enables both transmembrane and intracellular protein species to be easily transported to precise sub-cellular locations, ensuring the precision of targeted therapy. However, multiepitope mRNA vaccines also face significant challenges during the “folding” process from linear sequences to the correct three-dimensional structure, which is directly critical to the vaccine’s final efficacy. For example, if the polypeptides expressed by the vaccine fail to fold correctly, their conformation may significantly differ from that of the native antigen. This would render them ineffective for recognition by immune cells—akin to “a wrong key failing to unlock the door”—thereby preventing the activation of an immune response against the actual tumor and resulting in a loss of the vaccine’s immunogenicity (73). Furthermore, misfolded proteins are typically structurally unstable and more prone to rapid degradation by intracellular proteasomes. This means that even if the vaccine successfully expresses the antigen, it will be quickly “treated as waste” and cleared by the cell, failing to provide sufficiently sustained and potent immune stimulation (74).

### 4.2 Efficiency and low cost of mRNA vaccine production

Once fundamental production facilities and processes are established, adapting to different tumor antigens often requires only changing the mRNA sequence. This platform-based characteristic can, to a certain extent, save research, development, and production costs compared to other types of vaccines. Additionally, for traditional immunosuppressant therapies, the cost per treatment course can be as high as $11,733. For antibody-drug conjugates (ADCs), taking trastuzumab deruxtecan (DS-8201) as an example, the annual treatment cost in China reaches approximately 390,000 RMB. In contrast, it is projected that the annual treatment cost for universal mRNA cancer vaccines may be around $45,000.mRNA-based cancer immunotherapeutic agents employ mRNA to convey tumor-associated antigens or immunomodulatory molecular entities in conjunction with delivery vehicles and immunological adjuvants, aiming to evoke anti-neoplastic immune responses. The mRNA can be generated *ex vivo* by utilizing DNA templates, ribonucleoside triphosphates, and recombinant enzymatic substances (75, 76), during which a promoter of DNA-dependent RNA polymerase (such as T3, T7, or SP6) is integrated. The DNA template is subsequently linearized to act as a substrate for the synthesis of mRNA that is catalyzed by a DNA-dependent RNA polymerase. This is succeeded by template digestion by DNases. During the transcription process, a 5′ cap structure and a 3′ poly(A) tail are appended to boost translation efficiency in the *in vivo* environment. Subsequently, unbound nucleotides, enzymes, truncated RNA fragments, and remaining DNA residues are eliminated to purify the mRNA. This optimized procedure enables the expeditious production of mRNA, rendering it a desirable strategy for creating individualized cancer immunotherapies. Moreover, all the reagents and enzymes necessary to produce mRNA vaccines are commercially accessible. Notably, the manufacturing process of mRNA vaccines typically takes approximately 10 days, substantially shorter than that needed to produce other vaccine types (75). Therefore, the capacity to rapidly, cost-effectively, and readily scale up production significantly broadens the application prospects of mRNA-based vaccines for lung cancer.

### 4.3 mRNA vaccine safety

mRNA-based immunization endeavors to trigger or augment potent anti-tumor immune reactions. Synthetic mRNAs encoding TAAs or TSAs can be delivered through *ex vivo* mRNA-engineered autologous DCs or formulated or unformulated mRNA injections. However, safety remains the primary concern for any new treatment method. Notably, the manufacturing of *in vitro* transcribed mRNA employs a cell-free methodology, effectively circumventing risks of protein or viral contamination commonly observed in alternative vaccine development systems, such as replicating viral carriers, attenuated pathogens, and protein-based formulations. Furthermore, the swift intracellular conversion of mRNA transcripts into functional polypeptides substantially mitigates potential microbial contamination risks. In contrast to DNA-based immunization strategies, mRNA-based formulations avoid genomic integration events, thereby minimizing the likelihood of insertional mutagenesis and associated oncogenic risks. The inherent susceptibility of mRNA molecules to endogenous ribonuclease-mediated degradation enables precise temporal regulation of protein synthesis through half-life modulation strategies (77, 78).

Initial human trials evaluating unformulated mRNA administration in melanoma patients, initiated in 2008, established foundational safety and tolerability profiles for this therapeutic modality. Notably, no grade III or IV adverse reactions, as defined by the WHO, were observed during the trial (78). Encapsulated mRNA vaccines, such as those complexed with protamine and administered via intradermal delivery in advanced-stage malignancies, predominantly trigger localized injection-site inflammation or systemic asthenia. Furthermore, multicenter phase III studies utilizing standardized toxicity grading systems have validated that mRNA-based formulations exhibit favorable safety profiles and align with precision oncology frameworks through mechanism-driven therapeutic targeting (79–81).

Nevertheless, the potential adverse reactions from mRNA vaccine injections cannot be overlooked. As mentioned, part of the advantage of mRNA vaccines stems from the fact that even without adjuvants, the lipid nanoparticles (LNPs) containing mRNA can activate innate immune responses, exerting a pro-inflammatory effect and primarily inducing cytokines such as IL-1β and IL-6. However, a strong innate immune response is a double-edged sword: it is necessary to initiate subsequent adaptive immunity, but if overactivated, it can accelerate vaccine clearance and lead to systemic inflammatory reactions, such as common adverse effects like fever, chills, and fatigue. In preclinical studies, it has even been observed that mRNA vaccines encoding secreted IL-12, while enhancing anti-tumor effects, could trigger systemic inflammatory cytokine storms and significant toxicity such as weight loss (82). The precise mechanisms underlying mRNA vaccine-associated myocarditis remain under investigation. Notably, studies have found that in immune checkpoint inhibitor (ICI)-related myocarditis (irMyocarditis), there is a significant upregulation of programmed death-ligand 1 (PDL1) expression on cardiomyocytes. This suggests that certain immune-related adverse events may be linked to the excessive or abnormal activation of the immune system. Therefore, publicly available data from more participants are still needed to establish the safety profile of mRNA vaccines (83).

## 5 Research strategies for personalized lung cancer mRNA vaccines

**Figure 5:**
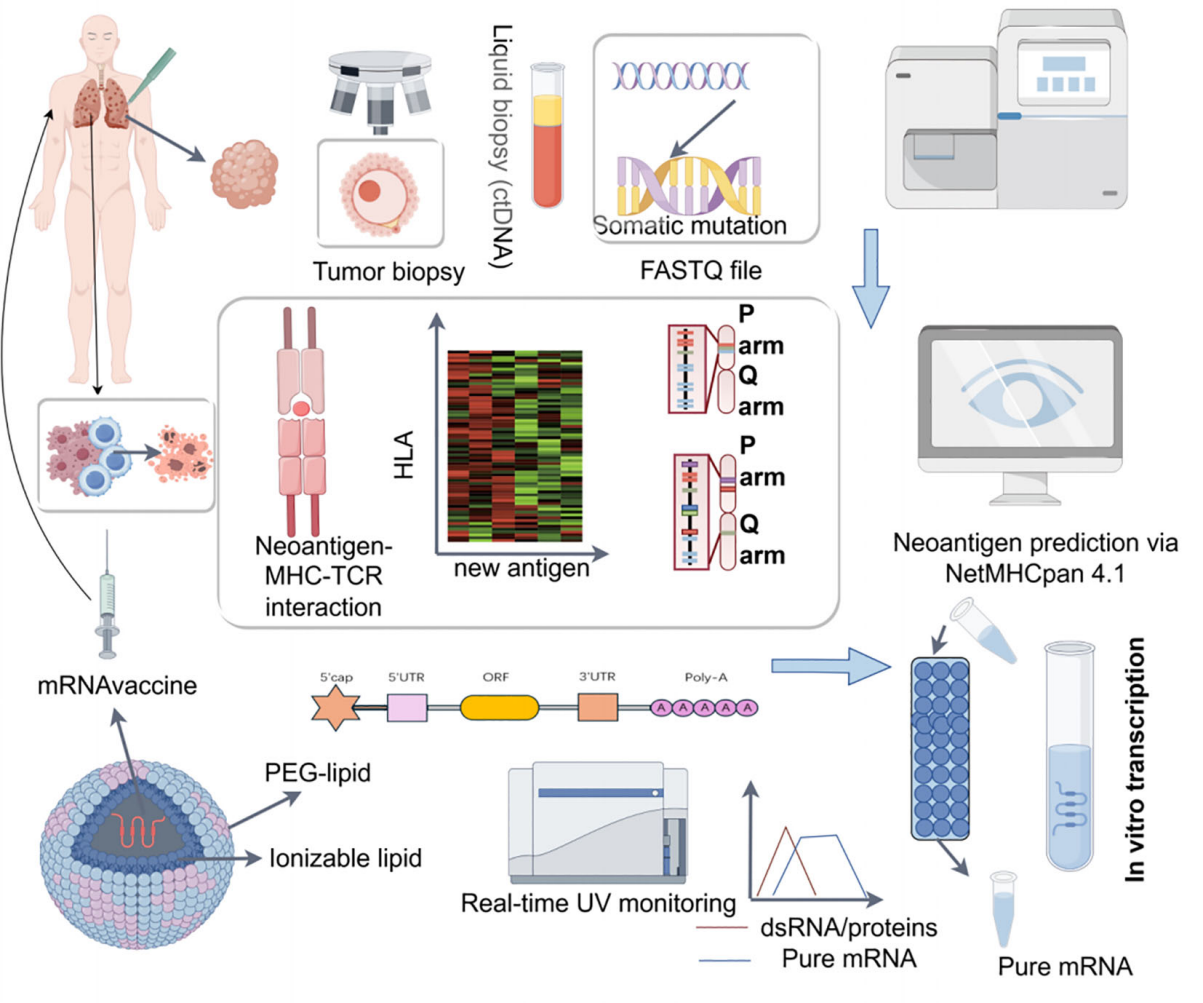
Workflow of personalized mRNA vaccine development for lung cancer. This schematic outlines the pipeline for constructing neoantigen-targeted mRNA vaccines. (1) Tumor biopsy or liquid biopsy-derived ctDNA is subjected to whole-exome/RNA sequencing to identify somatic mutations. (2) Neoantigen prediction using algorithms (e.g., NetMHC) prioritizes immunogenic epitopes. (3) Optimized mRNA sequences are synthesized with 5’ cap, nucleoside modifications (e.g., N1-methylpseudouridine), and extended poly-A tails to enhance stability and translation. (4) Lipid nanoparticle (LNP) encapsulation enables dendritic cell-targeted delivery. (5) Vaccines elicit tumor-specific T-cell responses post-administration. Key innovations include in silico neoantigen prioritization, nucleotide engineering to evade innate immunity, and ionizable lipid-based delivery systems.

mRNA vaccines, characterized by their precise targeting, relatively high production efficiency, reliable safety profile, and low economic cost, hold significant potential for personalized lung cancer treatment. Emerging evidence suggests that the development of personalized lung cancer mRNA vaccines should be divided into three key modules: identifying tumor antigens, constructing mRNA vaccines, and distinguishing immune subtypes (**Figure 5**).

### 5.1 Identification of lung cancer antigens

Antigen selection is the first crucial step in vaccine development. Ideal candidate vaccines should have tumor cell specificity, be involved in tumor occurrence and progression, and can evoke an immune response without immune tolerance and stimulate antitumor immunity (84–86). Immunogenic targets currently used for cancer treatment include TAAs and TSAs (86). TAAs typically show higher expression levels in tumor cells than in normal cells. However, as self-antigens, TAAs may induce immune tolerance, which can reduce the efficacy of vaccines. In contrast, TSAs are only found on cancer cells, have a high ability to trigger immune responses, and have many different epitopes, making them suitable for personalized vaccines. Creating customized mRNA vaccines for lung cancer involves identifying unique mutations in the tumor by analyzing advanced sequencing information derived from both tumor samples and corresponding normal tissues (87). Computational methods for predicting neoantigens are employed to assess the expression levels and forecast the binding affinity of peptides originating from mutated genes to MHC alleles. A high transcript expression level is strongly linked to more robust T-cell responses and can offset the low MHC binding affinity in some mutations (88). However, among the neoantigens predicted by computational tools, many fail to elicit effective T-cell immune responses *in vivo*. These false-positive neoantigens represent a major obstacle in advancing vaccine development. An important source of false positives is homologous sequences. If a neoantigen’s sequence is too similar to certain normal human proteins, to maintain self-tolerance, the corresponding T cells may have already been eliminated in the thymus (i.e., central tolerance) or are rendered anergic in the periphery. Therefore, it is essential to incorporate alignment against the human proteome sequence in the algorithm design to filter out candidate neoantigens with high similarity to self-sequences (89). Computationally predicted candidate neoantigens must undergo *in vitro* and *in vivo* experimental validation to confirm their true immunogenicity. Typically, the predicted neoantigen peptides are synthesized and co-cultured *in vitro* with immune cells (such as peripheral blood mononuclear cells, PBMCs) isolated from the patient’s blood. If the neoantigen can be recognized by specific T cells, it will activate and induce their proliferation. This can be assessed by detecting T-cell activation markers (e.g., CD137), measuring cytokine secretion (e.g., IFN-γ), or directly observing T-cell proliferation.

In addition to MHC-I-binding TSAs, further MHC-II-binding TSAs are essential for a successful antitumor immune response. Prediction tools, such as NetMHCpan and MHCflurry, are utilized to evaluate the interaction strength between ligands and MHC molecules (90–93). However, the stability of the neoepitope-MHC complex is a more critical factor for predicting immunogenicity than its binding affinity (93). NetMHCstabpan is a tool designed to predict the stability of these complexes, proving valuable in identifying immunogenic mutations (94). Beyond the mere display on the cell surface, the engagement between peptide-MHC complexes and T-cell receptors is vital for triggering immune responses. This engagement is predicted based on the interaction between the amino acid side chains of T cell receptors and the MHC-bound peptides (94). It is noteworthy that the expression and function of MHC molecules are finely regulated. For instance, recent studies have revealed that MHC class II molecules in murine antigen-presenting cells undergo branched ubiquitination mediated by the E3 ubiquitin ligase MARCH1, dependent on K11- and K63-linked chains. This modification regulates the endocytosis and degradation of MHC II, thereby influencing its antigen-presentation function. This suggests that beyond binding affinity, the metabolic regulation of MHC molecules themselves is a key factor affecting the efficiency of neoantigen presentation (95). In addition, tumors are not composed of identical cells; there exist genetic differences within them, known as tumor heterogeneity. This results in vaccine-targeted neoantigens possibly being present only in a subset of tumor cells (subclones), thereby granting a growth advantage to other cell clones that do not express these neoantigens (immune escape). The CalicoST algorithm, published in Nature Methods in 2024, provides a powerful tool to address this issue. It can infer allele-specific copy number variations from spatial transcriptomics (SRT) data and reconstruct the evolutionary trajectory of tumor clones within the tissue spatial context. This technology can identify genomic events that are difficult to detect with conventional methods, such as copy-number-neutral loss of heterozygosity, which may lead to the loss of neoantigens. By mapping the “evolutionary landscape” of the tumor, it can guide us to prioritize neoantigens present in the trunk clonal of all tumor cells as vaccine targets, thereby more effectively attacking the root of the tumor (96). Recently, promising pipelines for identifying tumor antigens have been established by screening overexpressed and mutated genes and identifying prognostic and APC-related candidates. These pipelines utilize sophisticated methods like next-generation sequencing and mass spectrometry to identify peptides bound to HLA and forecast neoantigens with precision (97). Currently, there is a lack of widely accepted, unified standard protocols and quality control measures for neoantigen prediction pipelines. Different research groups may employ different combinations of algorithms and parameters, making it difficult to compare and validate prediction results across platforms and studies. Despite progress in machine learning methods, there is still significant room for improvement in prediction accuracy. A considerable number of false-positive and false-negative predictions persist, as many candidate peptides identified computationally ultimately fail to elicit T-cell responses in *in vitro* experiments, further highlighting the limitations of current algorithms. Computational tools are typically based on relatively simplified models. For instance, current algorithms still struggle to fully integrate and simulate the impact of complex biological regulatory layers—such as the ubiquitination modification of MHC class II molecules—or the dynamic evolution of tumor spatial heterogeneity on the final presentation of neoantigens. In the future, more advanced and reliable algorithms and methods are certain to emerge, enabling the identification of highly effective antigens for personalized mRNA lung cancer vaccines (98).

However, although these methods are theoretically feasible, no corresponding validation measures have been established. Therefore, selecting the optimal antigens for mRNA vaccine development remains a significant challenge. However, recent advancements in computational biology have accelerated the prediction of tumor antigens. In the future, more advanced and reliable algorithms and methods will likely emerge, enabling the identification of effective antigens for personalized mRNA lung cancer vaccines.

### 5.2 Construction of lung cancer mRNA vaccines

Prior to the clinical implementation of mRNA-based cancer immunotherapies, several crucial aspects need to be resolved, such as the delivery mechanism, stability characteristics, translation efficiency, and immunogenic potential (23, 99, 100). For instance, unformulated mRNA, due to its large molecular dimensions, susceptibility to degradation, and electrical charge properties, cannot penetrate cell membranes with high efficiency and reach the cell cytoplasm. However, in the case of immature dendritic cells, which possess a unique ability to take in mRNA through micropinocytosis (99, 101). To improve mRNA delivery to APCs, the selection and optimization of mRNA formulations—such as liposomes, polymer complexes, polynucleosomes, and lipocomplexes—and administration routes are crucial. Once mRNA is successfully delivered, its duration of existence within the body must be precisely modulated since multiple elements impact the pharmacodynamic and pharmacokinetic features of mRNA-based treatments. To boost the resilience of the mRNA, its configuration ought to be enhanced, such as fine-tuning the 5′ cap structure, the poly(A) tail length, the untranslated regions’ sequences, and the protein-encoding ORF (102–104).

State-of-the-art mRNA vaccine delivery platforms predominantly utilize engineered nanoscale carrier architectures, incorporating biologically derived viral vectors and synthetic non-viral nanoparticles. These vectors not only trigger cellular immunity but also evoke humoral immune reactions. Lipid nanoparticles (LNPs) represent the principal nanomaterials for mRNA vaccine conveyance and usually comprise four constituents: ionizable cationic lipids, lipid-linked polyethylene glycol, cholesterol, and naturally occurring phospholipids. The production of LNPs represents a crucial procedure that has an immediate impact on their dimensions and encapsulation efficacy. The crux of creating minute and homogeneous LNPs lies in the swift combination of an excess amount of water and the ethanol-lipid phases. Microfluidic mixing, which uses an interdigitated herringbone structure, is an alternative technique for manufacturing LNPs across various scales (104). Once the mRNA vaccine enters the target cell, the formation of complexes with the released cationic lipids is crucial to enable the delivery of nucleic acid. The negatively charged lipids within the cell membrane can counteract the electrical charge of the cationic lipid vectors, disturbing the electrostatic forces between the lipid vectors and the nucleic acid molecules. This, in turn, eases the liberation of LNPs and the subsequent transfer of mRNA into the cytoplasmic region (105, 106). The extracellular stability of mRNA can be augmented through encapsulation within LNPs, as they safeguard it from breakdown by the omnipresent ribonucleases (107). The application of nanocarriers has the potential to prolong the lifespan of biologics. This is achieved through measures such as forestalling untimely release and decomposition, as well as evading elimination. The organs responsible for this potential elimination are the kidneys and the liver (108). Consequently, delivery systems founded on nanomaterials can preserve the extracellular stability of mRNA-based vaccines.

Notably, LNP-based mRNA vaccines can address the limitations of traditional carriers, such as suboptimal cellular protein synthesis, inadequate antigen payload, and issues related to APC maturation. These mRNA vaccines are independent of host influence and can induce a durable immune response.

Besides delivery and stability aspects, immunogenicity is also a factor that demands consideration. Immunogenicity is determined not only by the delivery and stability of mRNA but is also closely related to its translation efficiency. The higher the translation efficiency, the greater the quantity of antigen synthesized by the cells, thereby enabling a more robust activation of the immune response (109, 110). Accumulating data indicates an inhibitory feedback loop among mRNA and the immune response it induces. For example, exogenous RNA can stimulate the generation of type I IFNs through primary immune activation (111). Over-production of type I interferons restricts translational processes and accelerates the breakdown of ribosomal RNA and cellular mRNA (102). Specifically, exogenous RNA activates proteins such as PKR, OAS, and IFIT, which suppress translation efficiency by phosphorylating eIF2α and degrading RNA, thereby reducing the protein expression of mRNA. To overcome this challenge, optimizing the structure of mRNA—such as lengthening the poly(A) tail, optimizing the 5’ cap structure, modifying the mRNA sequence, and performing post-transcriptional modifications (e.g., using demethylation modifications)—can effectively reduce innate immune responses and enhance translation efficiency (112). For example, N¹-methyl-pseudouridine (m1Ψ) modification significantly reduces type I IFN activation and increases mRNA translation efficiency by 2- to 4-fold. An optimized poly(A) tail length can increase translation efficiency by 1.5-fold by improving the interaction between poly(A)-binding protein and eIF4G, thereby promoting translation (113, 114). eIF4E, as a key factor in mRNA translation, initiates the translation process by binding to the 5’ cap structure of mRNA (115). Optimizing the 5’ cap structure of mRNA to bind more efficiently with eIF4E can significantly enhance translation efficiency and increase antigen expression (116). Studies show that an optimized Cap1 cap structure can improve translation efficiency by 3- to 5-fold while enhancing mRNA stability and translation efficiency [Furuichi 2015]. These optimizations will directly increase the expression level of the target antigen, thereby enhancing immunogenicity and further promoting the generation of specific immune responses (117). Adding poly(A) tails, sequence modification, and post-transcriptional refinement can mitigate innate immune activation without altering mRNA translation (118–120). For example, The TriMix strategy enhances dendritic cell maturation and promotes immune responses through T-cell activation by encoding CD70, CD40L, and TLR4, demonstrating notable efficacy in lung cancer immunotherapy (121, 122). Specifically, CD70 and CD40L enhance T-cell immune activity via co-stimulation, while TLR4 activation further strengthens the ability of dendritic cells (DCs) to present tumor antigens via MHC-I molecules by boosting the NF-κB pathway. This enables mRNA-encoded antigens to more effectively activate specific T-cell responses (123, 124). These optimization strategies significantly enhance the immunogenicity of mRNA vaccines and improve their immunotherapeutic outcomes by precisely regulating mRNA translation efficiency and immune cell activation. Thus, optimizing mRNA vaccines requires not only improving delivery and stability but also enhancing immunogenicity through optimized translation efficiency, thereby more effectively boosting their therapeutic efficacy.

## 6 Conclusions and outlook

In summary, through multi-faceted optimization of antigen screening, vector engineering, and immunomodulation, mRNA vaccines are progressively overcoming existing technical barriers, offering a novel prospect for personalized immunotherapy in lung cancer (**Table 1**).

**Table 1:** Summary of Selected Clinical Trials Investigating mRNA-Based Neoantigen or Tumor-Associated Antigen Vaccines in Non-Small Cell Lung Cancer (NSCLC).

Official Title/ClinicalTrials.gov ID	Vaccine name	Target antigen	Study phase	study design	Outcome Measures
Clinical Trial on the Safety and Efficacy of Neoantigen Antigen mRNA Tumor Vaccine in the Treatment of Advanced Esophageal Cancer and Non-small Cell Lung Cancer **NCT03908671**	Personalized mRNA Tumor Vaccine	No publishing	Phase I	Open Label Single Group Assignment	1.Primary Outcome Measures:Number of participants with treatment-related adverse events as assessed by CTCAE v4.03 2.Secondary Outcome Measures: (1) Disease Control Rate (DCR)1.5 years (2)Progression-free Survival (PFS)2 years (3).Time to Tumor Progression (TTP)2 years (4).Overall Survival (OS)3 years
Combination Immunotherapy and Messenger Ribonucleic Acid (mRNA) Vaccine in Subjects With NSCLC **NCT03164772**	BI 1361849	**NY-ESO-1、MAGE-C2、MAGE-C1、survivin、5T4、** **MUC1**	Phase I and II	open-label, multicenter, 2-arm study	1.Primary Outcome Measures:Number of Subjects With Treatment-Emergent Adverse Events (TEAEs)(up to 15 months) 2.Secondary Outcome Measures:: (1)Median PFS by RECIST 1.1 as Estimated Using the Kaplan-Meier Method(up to 15 months) (2)Number of Subjects Without Progression at 8 and 24 Weeks by RECIST 1.1(up to 24 weeks) (3)Median PFS by irRECIST as Estimated Using the Kaplan-Meier Method(up to 15 months)
An Exploratory Study of Personalized Neoantigen mRNA Vaccine RGL-270 Combined with Adebrelimab in Non-Small Cell Lung Cancer Patients **NCT06685653**	RGL-270	Not applicable	phase IIT	open-label	1.Primary Outcome Measures: (1).The DLTs incidence of 21 days after first administration of RGL-270 in combination with Adebrelimab (2).Safety endpoints:90 days (± 7 days) after the last vaccine/adebelimab treatment or 30 days (±7 days) after the last chemotherapy Secondary Outcome Measures: (1)Change from baseline of LNPs(18 months/24 months after the first dose) 2.Change from baseline of neoantigen-specific T cell(18 months/24 months after the frst dose) (3)Change from baseline of ADA(18 months/24 months after the frst dose) 4.The DFS, OS, DFS rate and OS rate in Resectable NSCLC(18 months/24 months after the frst dose)
A Study of mRNA-5671/​V941 as Monotherapy and in Combination With Pembrolizumab **NCT03948763**	mRNA-5671/V941	**G12V、G13D、G12C**	phase I	Open-Label, Multicenterl	1.Primary Outcome Measures: (1)Dose-Limiting Toxicities (DLTs)-Cycle 1 (Up to 21 days) (2)Number of Participants Who Experienced an Adverse Event (AE)-Up to approximately 27 months (3)Number of Participants Who Discontinued Study Treatment Due to an AE-Up to approximately 24 months 2.Secondary Outcome Measures: (1)Objective Response Rate (ORR)(Up to approximately 24 months) (2Presence of Mutant Kirsten Rat Sarcoma (KRAS) Specific T Cells)(Cycle 1 - Cycle 9 (a cycle is 3 weeks) and at the Discontinuation Visit (at the time of withdrawal or up to 30 weeks, whichever occurs first)) (3Mean Change From Baseline in Quantity of Mutant KRAS Specific T Cells(Baseline and Cycle 1 - Cycle 9 (a cycle is 3 weeks) and at the Discontinuation Visit (at the time of withdrawal or up to 30 weeks, whichever occurs first)))
An Exploratory Study of the Personalized MRNA Neoantigen Vaccine in Combination with Adebrelimab As Adjuvant Treatment for Patients with Resected Non-small Cell Lung Cancer **NCT06735508**	mRNA Neoantigen Vaccine	No publishing	Early Phase 1	an open, prospective, exploratory clinical trial	1.Primary Outcome Measures: (1)safety and tolerability(From the start of study treatments until 30 days after last dose of study treatments.) (2)MTD or MAD(From the start of study treatments until the end of the safety run-in phase, about 4 months.) 2.Secondary Outcome Measures: (1)immunogenicity:(From the sugery til the end of study treatment,about 18 months.) (2)Preliminary efficacy(3years)
Clinical Trial Evaluating the Safety, Tolerability and Preliminary Efficacy of BNT116 Alone and in Combinations in Patients With Advanced Non-small Cell Lung Cancer (LuCa-MERIT-1) **NCT05142189**	BNT116	**MAGE A3、CLDN6、KK-LC-1、PRAME、MAGE A4** **MAGE C1**	Phase I	Open Label Non-Randomized	1.Primary Outcome Measures: (1)Cohorts 1, 2, 3, 4, 6, and 7: Occurrence of dose-limiting toxicities (DLTs) during Cycle 1 assessed during the first cycle (21 days) (2)Cohorts 1 to 7: Occurrence of treatment-emergent adverse events (TEAEs) reported by relationship, seriousness, and grade according to National Cancer Institute-Common Terminology Criteria for Adverse Events (NCI-CTCAE) v5.0 up to 27 months... 2.Secondary Outcome Measures:: (1) Cohorts 1, 2, 3, 4, and 7: Overall response rate (ORR) defined as the number of patients with complete response (CR) or partial response (PR) as best overall response (BOR)up to 27 months...
A Study of V940 Plus Pembrolizumab (MK-3475) Versus Placebo Plus Pembrolizumab in Participants With Non-small Cell Lung Cancer (V940-002) (INTerpath-002) **NCT06077760**	V940	No publishing	Phase III	Open Label Randomized, Double-blind, Placebo- and Active-Comparator-Controlled Clinical Study	1.Primary Outcome Measures: Disease- Free Survival (DFS)(Up to ~78 months) 2.Secondary Outcome Measures:: (1)Overall Survival (OS)(Up to ~12 years) (2)Distant Metastasis-Free Survival (DMFS(Up to ~12 years) (3)Lung Cancer Specific Survival (LCSS)(Up to ~12 years) (4)Change from Baseline in the European Organization for Research and Treatment of Cancer (EORTC)-Quality of Life Questionnaire-Core 30 (QLQ-C30) Global Health Status/Quality of Life (Items 29 and 30) Combined Score(Baseline and up to ~12 years)

Text in blue denotes ClinicalTrials.gov registry numbers (e.g., NCT03908671), while text in red denotes publicly disclosed and known target antigens.

mRNA vaccines, leveraging their advantages in rapid development, controllable cost, and high personalization, provide a new direction for personalized lung cancer treatment. Their strengths in development speed, controllable production cost, and personalized design demonstrate unique potential compared to traditional vaccine platforms. Preclinical studies and early clinical trials (such as BI 1361849 and CV 9201) have preliminarily confirmed the safety of mRNA vaccines and their ability to elicit tumor-specific immune responses. In the study by Papachristofilou et al., the number of functional CD4^+^ and/or CD8^+^ T lymphocytes increased to more than twice the pre-treatment level in 40% of patients after vaccination. Sebastian’s team also confirmed the favorable tolerability of CV 9201 in patients with advanced NSCLC. Particularly when combined with local radiotherapy or immune checkpoint inhibitors, mRNA vaccines show a trend of synergistic enhancement, as evidenced by the ongoing phase I/II clinical trial of BI 1361849 combined with durvalumab and tremelimumab (NCT03164772).

However, while acknowledging these positive advances, we must soberly recognize that personalized mRNA vaccines have not yet achieved definitive validation of clinical efficacy in lung cancer treatment, and their translation from the laboratory to widespread clinical application still faces multiple fundamental obstacles. Firstly, the precision of target selection remains a core challenge. Currently, most candidate vaccines still rely on tumor-associated antigens, and their inherent immune tolerance limits therapeutic breakthroughs. Neoantigens based on tumor mutations are theoretically more advantageous, but their identification faces challenges such as high false-positive prediction rates, complex experimental validation processes, and tumor heterogeneity leading to immune escape driven by subclonal antigens. These factors collectively make it difficult to stably screen highly effective and broadly applicable immunogenic targets.

Secondly, the suppressive tumor immune microenvironment presents another major obstacle. Even if a vaccine successfully activates antigen-specific T cells, these immune effector cells may become functionally exhausted within the tumor locale due to immune checkpoint signals, inhibitory cytokine networks, and infiltrating immunosuppressive cells, thereby hindering effective tumor cell killing. This explains why monotherapy regimens effective in preclinical models often show limited efficacy in human clinical trials.

Notably, combination therapeutic strategies are considered a key path to overcoming the aforementioned bottlenecks. As an “igniter” of the immune system, the combination of mRNA vaccines with immune checkpoint inhibitors (e.g., anti-PD-1/PD-L1 antibodies) is theoretically complementary: the vaccine activates and expands the tumor-specific T-cell repertoire, while the checkpoint inhibitor relieves the suppressive state of the tumor microenvironment, thereby synergistically enhancing the quality and durability of the antitumor immune response. Beyond immune checkpoint inhibitors, exploration of various administration routes also merits attention. For instance, the locally administered CLPP/mIL-15 complex has shown antitumor activity in preclinical lung metastasis models, suggesting that combining local intratracheal administration with systemic delivery may enhance the precision of lung cancer treatment.

Looking ahead, breakthroughs in this field will depend on: establishing more accurate neoantigen prediction and validation systems; developing novel delivery systems that efficiently target lymphoid organs and overcome immune suppression; and rigorously validating combination regimens of mRNA vaccines with other therapies (especially immune checkpoint inhibitors and radiotherapy) in larger-scale clinical trials. The true value of mRNA vaccines may lie not as a monotherapy, but as a key component within a combinatorial cancer immunotherapy strategy. Through multidisciplinary collaboration and continuous technological innovation, mRNA vaccines hold the potential to ultimately become a crucial force in improving outcomes for lung cancer patients. However, further research is still needed to determine whether they can evolve into a reliable, effective, and potentially independent treatment modality.

## References

[1] Seegobin K Majeed U Wiest N Manochakian R Lou Y Zhao Y . Immunotherapy in non-small cell lung cancer with actionable mutations other than EGFR. Front Oncol. (2021) 11:750657. doi: 10.3389/fonc.2021.750657, PMID: 34926258 PMC8671626

[2] Li Q Yuan D Ma C Liu Y Ma L Lv T . A new hope: the immunotherapy in small cell lung cancer. Neoplasma. (2016) 63:342–50. doi: 10.4149/302_151001N511, PMID: 26925794

[3] Boloker G Wang C Zhang J . Updated statistics of lung and bronchus cancer in United States (2018). J Thorac Dis. (2018) 10:1158–61. doi: 10.21037/jtd.2018.03.15, PMID: 29708136 PMC5906235

[4] Mamdani H Matosevic S Khalid AB Durm G Jalal SI . Immunotherapy in lung cancer: current landscape and future directions. Front Immunol. (2022) 13:823618. doi: 10.3389/fimmu.2022.823618, PMID: 35222404 PMC8864096

[5] Ercan D Zejnullahu K Yonesaka K Xiao Y Capelletti M Rogers A . Amplification of EGFR T790M causes resistance to an irreversible EGFR inhibitor. Oncogene. (2010) 29:2346–56. doi: 10.1038/onc.2009.526, PMID: 20118985 PMC2859699

[6] Lahiri A Maji A Potdar PD Singh N Parikh P Bisht B . Lung cancer immunotherapy: progress, pitfalls, and promises. Mol Cancer. (2023) 22:40. doi: 10.1186/s12943-023-01740-y, PMID: 36810079 PMC9942077

[7] Tartarone A Lerose R . Clinical approaches to treat patients with non-small cell lung cancer and epidermal growth factor receptor tyrosine kinase inhibitor acquired resistance. Ther Adv Respir Dis. (2015) 9:242–50. doi: 10.1177/1753465815587820, PMID: 26016841

[8] Chong CR Jänne PA . The quest to overcome resistance to EGFR-targeted therapies in cancer. Nat Med. (2013) 19:1389–400. doi: 10.1038/nm.3388, PMID: 24202392 PMC4049336

[9] Malhotra J Jabbour SK Aisner J . Current state of immunotherapy for non-small cell lung cancer. Transl Lung Cancer Res. (2017) 6:196–211. doi: 10.21037/tlcr.2017.03.01, PMID: 28529902 PMC5420529

[10] Pardoll DM . The blockade of immune checkpoints in cancer immunotherapy. Nat Rev Cancer. (2012) 12:252–64. doi: 10.1038/nrc3239, PMID: 22437870 PMC4856023

[11] Pardoll DM . Cancer and the immune system: basic concepts and targets for intervention. Semin Oncol. (2015) 42:523–38. doi: 10.1053/j.seminoncol.2015.05.003, PMID: 26320058 PMC5595144

[12] Gang X Yan J Li X Shi S Xu L Liu R . Immune checkpoint inhibitors rechallenge in non-small cell lung cancer: Current evidence and future directions. Cancer Lett. (2024) 604:217241. doi: 10.1016/j.canlet.2024.217241, PMID: 39260670

[13] Besse B Felip E Garcia Campelo R Cobo M Mascaux C Madroszyk A . ATALANTE-1 study group. Randomized open-label controlled study of cancer vaccine OSE2101 versus chemotherapy in HLA-A2-positive patients with advanced non-small-cell lung cancer with resistance to immunotherapy: ATALANTE-1. Ann Oncol. (2023) 34:920–33. doi: 10.1016/j.annonc.2023.07.006, PMID: 37704166

[14] Jardim DL Goodman A de Melo Gagliato D Kurzrock R . The challenges of tumor mutational burden as an immunotherapy biomarker. Cancer Cell. (2021) 39:154–73. doi: 10.1016/j.ccell.2020.10.001, PMID: 33125859 PMC7878292

[15] Sun S Liu L Zhang J Sun L Shu W Yang Z . The role of neoantigens and tumor mutational burden in cancer immunotherapy: advances, mechanisms, and perspectives. J Hematol Oncol. (2025) 18:84. doi: 10.1186/s13045-025-01732-z, PMID: 40898324 PMC12406617

[16] Lu D Chen Y Jiang M Wang J Li Y Ma K . KRAS G12V neoantigen specific T cell receptor for adoptive T cell therapy against tumors. Nat Commun. (2023) 14:6389. doi: 10.1038/s41467-023-42010-1, PMID: 37828002 PMC10570350

[17] Chen J Ye Z Huang C Qiu M Song D Li Y . Lipid nanoparticle-mediated lymph node-targeting delivery of mRNA cancer vaccine elicits robust CD8+ T cell response. Proc Natl Acad Sci U S A. (2022) 119:e2207841119. doi: 10.1073/pnas.2207841119, PMID: 35969778 PMC9407666

[18] Zhang H Liu H Xu Y Huang H Liu Y Wang J . Deep generative models design mRNA sequences with enhanced translational capacity and stability. Science. (2025) 390:eadr8470. doi: 10.1126/science.adr8470, PMID: 40875799

[19] Atmaca A Alt J Baz DV Dziadziuszko R Moreno V Pose V . 1486 preliminary results from LuCa-MERIT-1, a phase I trial evaluating BNT116, a fixed antigen mRNA vaccine, plus Cemiplimab in advanced non-small cell lung Cancer after progression on PD-1 inhibition. In: Proceedings of the late-breaking. London, UK: BMJ Publishing Group Ltd (2024). p. A1716–6.

[20] Ding Z Li Q Zhang R Xie L Shu Y Gao S . Personalized neoantigen pulsed dendritic cell vaccine for advanced lung cancer. Signal Transduct Target Ther. (2021) 6:26. doi: 10.1038/s41392-020-00448-5, PMID: 33473101 PMC7817684

[21] Moore ZS Seward JF Lane JM . Smallpox. Lancet. (2006) 367:425–35. doi: 10.1016/S0140-6736(06)68143-9, PMID: 16458769

[22] Bangham AD Standish MM Watkins JC Weissmann G . The diffusion of ions from a phospholipid model membrane system. Protoplasma. (1967) 63:183–7., PMID: 6037197

[23] Deamer DW . From “banghasomes” to liposomes: a memoir of Alec Bangham, 1921–2010. FASEB J. (2010) 24:1308–10. doi: 10.1096/fj.10-0503, PMID: 20430797

[24] Gregoriadis G . The carrier potential of liposomes in biology and medicine (second of two parts). N Engl J Med. (1976) 295:765–70. doi: 10.1056/NEJM197609302951406, PMID: 785256

[25] Malone RW Felgner PL Verma IM . Cationic liposome-mediated RNA transfection. Proc Natl Acad Sci U S A. (1989) 86:6077–81. doi: 10.1073/pnas.86.16.6077, PMID: 2762315 PMC297778

[26] Jirikowski GF Sanna PP Maciejewski-Lenoir D Bloom FE . Reversal of diabetes insipidus in Brattleboro rats: intrahypothalamic injection of vasopressin mRNA. Science. (1992) 255:996–8. doi: 10.1126/science.1546298, PMID: 1546298

[27] Hussain MS Sultana A Bisht AS Gupta G . Groundbreaking mRNA lung cancer vaccine trials: A new dawn in cancer treatment. Curr Cancer Drug Targets. (2025) 25:962–7. doi: 10.2174/0115680096360059250131075456, PMID: 39957693

[28] Weber JS Carlino MS Khattak A Meniawy T Ansstas G Taylor MH . Individualised neoantigen therapy mRNA-4157 (V940) plus pembrolizumab versus pembrolizumab monotherapy in resected melanoma (KEYNOTE-942): a randomised, phase 2b study. Lancet. (2024) 403:632–44. doi: 10.1016/S0140-6736(23)02268-7, PMID: 38246194

[29] Sahin U Derhovanessian E Miller M Kloke BP Simon P Löwer M . Personalized RNA mutanome vaccines mobilize poly-specific therapeutic immunity against cancer. Nature. (2017) 547:222–6. doi: 10.1038/nature23003, PMID: 28678784

[30] Salaberry L Covo-Vergara Á Beloki U Silva-Pilipich N Smerdou C . Minimalist mRNAs: A fast track to cancer vaccines. Mol Ther Nucleic Acids. (2025) 36:102669. doi: 10.1016/j.omtn.2025.102669, PMID: 40896591 PMC12395492

[31] Alberer M Gnad-Vogt U Hong HS Mehr KT Backert L Finak G . Safety and immunogenicity of a mRNA rabies vaccine in healthy adults: an open-label, non-randomised, prospective, first-in-human phase 1 clinical trial. Lancet. (2017) 390:1511–20. doi: 10.1016/S0140-6736(17)31665-3, PMID: 28754494

[32] Schnee M Vogel AB Voss D Petsch B Baumhof P Kramps T . An mRNA vaccine encoding rabies virus glycoprotein induces protection against lethal infection in mice and correlates of protection in adult and newborn pigs. PLoS Negl Trop Dis. (2016) 10:e0004746. doi: 10.1371/journal.pntd.0004746, PMID: 27336830 PMC4918980

[33] Lundstrom K . Replicon RNA viral vectors as vaccines. Vaccines (Basel). (2016) 4:39. doi: 10.3390/vaccines4040039, PMID: 27827980 PMC5192359

[34] Casmil IC Jin J Won EJ Huang C Liao S Cha-Molstad H . The advent of clinical self-amplifying RNA vaccines. Mol Ther. (2025) 33:2565–82. doi: 10.1016/j.ymthe.2025.03.060, PMID: 40186353 PMC12172325

[35] Wang Y Zhang Z Luo J Han X Wei Y Wei X . mRNA vaccine: a potential therapeutic strategy. Mol Cancer. (2021) 20:33. doi: 10.1186/s12943-021-01311-z, PMID: 33593376 PMC7884263

[36] Carvalho T . Personalized anti-cancer vaccine combining mRNA and immunotherapy tested in melanoma trial. Nat Med. (2023) 29:2379–80. doi: 10.1038/d41591-023-00072-0, PMID: 37773210

[37] Lorentzen CL Haanen JB Met Ö Svane IM . Clinical advances and ongoing trials on mRNA vaccines for cancer treatment. Lancet Oncol. (2022) 23:e450–e8. doi: 10.1016/S1470-2045(22)00608-8, PMID: 36174631 PMC9512276

[38] Evans R Lee K Wallace PK Reid M Muhitch J Dozier A . Augmenting antibody response to EGF-depleting immunotherapy: Findings from a phase I trial of CIMAvax-EGF in combination with nivolumab in advanced stage NSCLC. Front Oncol. (2022) 12:958043. doi: 10.3389/fonc.2022.958043, PMID: 35992783 PMC9382666

[39] Rodriguez PC Popa X Martínez O Mendoza S Santiesteban E Crespo T . A phase III clinical trial of the epidermal growth factor vaccine CIMAvax-EGF as switch maintenance therapy in advanced non-small cell lung cancer patients. Clin Cancer Res. (2016) 22:3782–90. doi: 10.1158/1078-0432.CCR-15-0855, PMID: 26927662

[40] Sullenger BA Nair S . From the RNA world to the clinic. Science. (2016) 352:1417–20. doi: 10.1126/science.aad8709, PMID: 27313039 PMC6035743

[41] Kirschman JL Bhosle S Vanover D Blanchard EL Loomis KH Zurla C . Characterizing exogenous mRNA delivery, trafficking, cytoplasmic release and RNA-protein correlations at the level of single cells. Nucleic Acids Res. (2017) 45:e113. doi: 10.1093/nar/gkx290, PMID: 28449134 PMC5499550

[42] Chiou SH Tseng D Reuben A Mallajosyula V Molina IS Conley S . Global analysis of shared T cell specificities in human non-small cell lung cancer enables HLA inference and antigen discovery. Immunity. (2021) 54:586–602.e8. doi: 10.1016/j.immuni.2021.02.014, PMID: 33691136 PMC7960510

[43] Butts C Maksymiuk A Goss G Soulières D Marshall E Cormier Y . Updated survival analysis in patients with stage IIIB or IV non-small-cell lung cancer receiving BLP25 liposome vaccine (L-BLP25): phase IIB randomized, multicenter, open-label trial. J Cancer Res Clin Oncol. (2011) 137:1337–42. doi: 10.1007/s00432-011-1003-3, PMID: 21744082 PMC11828286

[44] Saavedra D Crombet T . CIMAvax-EGF: a new therapeutic vaccine for advanced non-small cell lung cancer patients. Front Immunol. (2017) 8:269. doi: 10.3389/fimmu.2017.00269, PMID: 28348561 PMC5346887

[45] Vansteenkiste JF Cho BC Vanakesa T De Pas T Zielinski M Kim MS . Efficacy of the MAGE-A3 cancer immunotherapeutic as adjuvant therapy in patients with resected MAGE-A3-positive non-small-cell lung cancer (MAGRIT): a randomised, double-blind, placebo-controlled, phase 3 trial. Lancet Oncol. (2016) 17:822–35. doi: 10.1016/S1470-2045(16)00099-1, PMID: 27132212

[46] Awakoaiye B Li S Sanchez S Dangi T Irani N Arroyo L . Comparative analysis of adenovirus, mRNA, and protein vaccines reveals context-dependent immunogenicity and efficacy. JCI Insight. (2025) 10:e198069. doi: 10.1172/jci.insight.198069, PMID: 41212056 PMC12643482

[47] Raina D Kosugi M Ahmad R Panchamoorthy G Rajabi H Alam M . Dependence on the MUC1-C oncoprotein in non–small cell lung cancer cells. Mol Cancer Ther. (2011) 10:806–16. doi: 10.1158/1535-7163.MCT-10-1050, PMID: 21421804 PMC3092019

[48] Study details, in: Vaccine therapy in treating patients with stage IIIB, stage IV, or recurrent non-small cell lung cancer. ClinicalTrials.gov. Available online at: https://clinicaltrials.gov/study/NCT00601094?cond=NCT00601094%C2%A0&rank=1 (Accessed November 30, 2024).

[49] Lee JM Lee MH Garon E Goldman JW Salehi-Rad R Baratelli FE . Phase I trial of intratumoral injection of CCL21 gene–modified dendritic cells in lung cancer elicits tumor-specific immune responses and CD8+ T-cell infiltration. Clin Cancer Res. (2017) 23:4556–68. doi: 10.1158/1078-0432.CCR-16-2821, PMID: 28468947 PMC5599263

[50] Yu J Sun H Cao W Song X Li J Han M . Research progress on dendritic cell vaccines in cancer immunotherapy. Exp Hematol Oncol. (2022) 11:3. doi: 10.1186/s40164-022-00257-2, PMID: 35074008 PMC8784280

[51] Xu Y Wang M . Progress in immunotherapy for non-small cell lung cancer. Zhongguo Fei Ai Za Zhi. (2014) 17:34–41. doi: 10.3779/j.issn.1009-3419.2014.01.06, PMID: 24398312 PMC6000202

[52] Lundstrom K . Self-amplifying RNA viruses as RNA vaccines. Int J Mol Sci. (2020) 21:5130. doi: 10.3390/ijms21145130, PMID: 32698494 PMC7404065

[53] Limacher JM Quoix E . TG4010: A therapeutic vaccine against MUC1 expressing tumors. OncoImmunology. (2012) 1:791–2. doi: 10.4161/onci.19863, PMID: 22934285 PMC3429597

[54] Ramlau R Quoix E Rolski J Pless M Lena H Lévy E . A phase II study of Tg4010 (Mva-Muc1-Il2) in association with chemotherapy in patients with stage III/IV non-small cell lung cancer. J Thorac Oncol. (2008) 3:735–44. doi: 10.1097/JTO.0b013e31817c6b4f, PMID: 18594319

[55] Sellars MC Wu CJ Fritsch EF . Cancer vaccines: building a bridge over troubled waters. Cell. (2022) 185:2770–88. doi: 10.1016/j.cell.2022.06.035, PMID: 35835100 PMC9555301

[56] Verbeke R Hogan MJ Loré K Pardi N . Innate immune mechanisms of mRNA vaccines. Immunity. (2022) 55:1993–2005. doi: 10.1016/j.immuni.2022.10.014, PMID: 36351374 PMC9641982

[57] Shoja Doost J Fazel F Boodhoo N Sharif S . mRNA vaccination: an outlook on innate sensing and adaptive immune responses. Viruses. (2024) 16:1404. doi: 10.3390/v16091404, PMID: 39339880 PMC11437395

[58] Cai C Tang YD Xu G Zheng C . The crosstalk between viral RNA- and DNA-sensing mechanisms. Cell Mol Life Sci. (2021) 78:7427–34. doi: 10.1007/s00018-021-04001-7, PMID: 34714359 PMC8554519

[59] Teijaro JR Farber DL . COVID-19 vaccines: modes of immune activation and future challenges. Nat Rev Immunol. (2021) 21:195–7. doi: 10.1038/s41577-021-00526-x, PMID: 33674759 PMC7934118

[60] Weidensee B Sahu I . Decrypting the immune symphony for RNA vaccines. Vaccines (Basel). (2025) 13:882. doi: 10.3390/vaccines13080882, PMID: 40872966 PMC12390541

[61] Manfrini N Notarbartolo S Grifantini R Pesce E . SARS-coV-2: A glance at the innate immune response elicited by infection and vaccination. Antibodies (Basel). (2024) 13:13. doi: 10.3390/antib13010013, PMID: 38390874 PMC10885122

[62] Zhang C Stewart W Teng Y Hu B Xu X Zhang XQ . mRNA-based vaccination drives *in vivo* dendritic cell reprogramming and selective cytotoxic T lymphocyte modulation for enhanced antitumor immunity. ACS Nano. (2025) 19:38267–83. doi: 10.1021/acsnano.5c09365, PMID: 41151069

[63] Clemente B Denis M Silveira CP Schiavetti F Brazzoli M Stranges D . Straight to the point: targeted mRNA-delivery to immune cells for improved vaccine design. Front Immunol. (2023) 14:1294929. doi: 10.3389/fimmu.2023.1294929, PMID: 38090568 PMC10711611

[64] Wei J Hui AM . The paradigm shift in treatment from Covid-19 to oncology with mRNA vaccines. Cancer Treat Rev. (2022) 107:102405. doi: 10.1016/j.ctrv.2022.102405, PMID: 35576777 PMC9068246

[65] Gote V Bolla PK Kommineni N Butreddy A Nukala PK Palakurthi SS . A comprehensive review of mRNA vaccines. Int J Mol Sci. (2023) 24:2700. doi: 10.3390/ijms24032700, PMID: 36769023 PMC9917162

[66] Rijkers GT Weterings N Obregon-Henao A Lepolder M Dutt TS van Overveld FJ . Antigen presentation of mRNA-based and virus-vectored SARS-coV-2 vaccines. Vaccines (Basel). (2021) 9:848. doi: 10.3390/vaccines9080848, PMID: 34451973 PMC8402319

[67] Ling J Chen H Huang M Wang J Du X . An mRNA vaccine encoding proteasome-targeted antigen enhances CD8+ T cell immunity. J Control Release. (2025) 381:113578. doi: 10.1016/j.jconrel.2025.02.074, PMID: 40015339

[68] Chen K Hu J Li J Wu G Tie X Wu H . Harnessing cellular immunity for next-generation vaccines against respiratory viruses: mechanisms, platforms, and optimization strategies. Front Immunol. (2025) 16:1618406. doi: 10.3389/fimmu.2025.1618406, PMID: 40881694 PMC12380765

[69] Noor R . mRNA vaccines as an efficient approach for the rapid and robust induction of host immunity against SARS-coV-2. SN Compr Clin Med. (2022) 4:88. doi: 10.1007/s42399-022-01168-3, PMID: 35402783 PMC8975617

[70] Roche PA Furuta K . The ins and outs of MHC class II-mediated antigen processing and presentation. Nat Rev Immunol. (2015) 15:203–16. doi: 10.1038/nri3818, PMID: 25720354 PMC6314495

[71] Nguyen CM Vu TT Nguyen M Ho DQ Le TH Tran HTN . Neoantigen-based mRNA vaccine exhibits superior anti-tumor activity compared to synthetic long peptides in an *in vivo* lung carcinoma model. Cancer Immunol Immunother. (2025) 74:145. doi: 10.1007/s00262-025-03992-7, PMID: 40072566 PMC11949242

[72] Ma S Li X Mai Y Guo J Zuo W Yang J . Immunotherapeutic treatment of lung cancer and bone metastasis with a mPLA/mRNA tumor vaccine. Acta Biomater. (2023) 169:489–99. doi: 10.1016/j.actbio.2023.07.059, PMID: 37536492

[73] Corral-Lugo A López-Siles M López D McConnell MJ Martin-Galiano AJ . Identification and analysis of unstructured, linear B-cell epitopes in SARS-coV-2 virion proteins for vaccine development. Vaccines (Basel). (2020) 8:397. doi: 10.3390/vaccines8030397, PMID: 32698423 PMC7564417

[74] Shah M Rafiq S Woo HG . Challenges and considerations in multi-epitope vaccine design surrounding toll-like receptors. Trends Pharmacol Sci. (2024) 45:1104–18. doi: 10.1016/j.tips.2024.10.013, PMID: 39603961

[75] Weng TY Yen MC Huang CT Hung JJ Chen YL Chen WC . DNA vaccine elicits an efficient antitumor response by targeting the mutant Kras in a transgenic mouse lung cancer model. Gene Ther. (2014) 21:888–96. doi: 10.1038/gt.2014.67, PMID: 25077772

[76] Lopes A Vandermeulen G Préat V . Cancer DNA vaccines: current preclinical and clinical developments and future perspectives. J Exp Clin Cancer Res. (2019) 38:146. doi: 10.1186/s13046-019-1154-7, PMID: 30953535 PMC6449928

[77] Ott PA Hu Z Keskin DB Shukla SA Sun J Bozym DJ . An immunogenic personal neoantigen vaccine for patients with melanoma. Nature. (2017) 547:217–21. doi: 10.1038/nature22991, PMID: 28678778 PMC5577644

[78] Pardi N Hogan MJ Porter FW Weissman D . mRNA vaccines — a new era in vaccinology. Nat Rev Drug Discov. (2018) 17:261–79. doi: 10.1038/nrd.2017.243, PMID: 29326426 PMC5906799

[79] Jackson NAC Kester KE Casimiro D Gurunathan S DeRosa F . The promise of mRNA vaccines: a biotech and industrial perspective. NPJ Vaccines. (2020) 5:11. doi: 10.1038/s41541-020-0159-8, PMID: 32047656 PMC7000814

[80] Pardi N Muramatsu H Weissman D Karikó K . *In vitro* Transcription of long RNA Containing modified nucleosides. Methods Mol Biol. (2013) 969:29–42. doi: 10.1007/978-1-62703-260-5_2, PMID: 23296925

[81] Kauffman KJ Webber MJ Anderson DG . Materials for non-viral intracellular delivery of messenger RNA therapeutics. J Control Release. (2016) 240:227–34. doi: 10.1016/j.jconrel.2015.12.032, PMID: 26718856

[82] Aunins EA Phan AT Alameh MG Dwivedi G Cruz-Morales E Christian DA . An Il12 mRNA-LNP adjuvant enhances mRNA vaccine-induced CD8 T cell responses. Sci Immunol. (2025) 10:eads1328. doi: 10.1126/sciimmunol.ads1328, PMID: 40478935 PMC13012528

[83] Li Y Li X Liu F Zhang H Guo J Liu G . Targeting ligand PDL1 for cardiotoxicity assessment and cardiac protection in immune-related myocarditis. Nanomed (Lond). (2025) 27:1–17. doi: 10.1080/17435889.2025.2595118, PMID: 41306058 PMC12785222

[84] Karikó K Muramatsu H Welsh FA Ludwig J Kato H Akira S . Incorporation of pseudouridine into mrna yields superior nonimmunogenic vector with increased translational capacity and biological stability. Mol Ther. (2008) 16:1833–40. doi: 10.1038/mt.2008.200, PMID: 18797453 PMC2775451

[85] Weide B Carralot JP Reese A Scheel B Eigentler TK Hoerr I . Results of the first phase I/II clinical vaccination trial with direct injection of mRNA. J Immunother. (2008) 31:180–8. doi: 10.1097/CJI.0b013e31815ce501, PMID: 18481387

[86] Beck JD Reidenbach D Salomon N Sahin U Türeci Ö Vormehr M . mRNA therapeutics in cancer immunotherapy. Mol Cancer. (2021) 20:69. doi: 10.1186/s12943-021-01348-0, PMID: 33858437 PMC8047518

[87] Heine A Juranek S Brossart P . Clinical and immunological effects of mRNA vaccines in Malignant diseases. Mol Cancer. (2021) 20:52. doi: 10.1186/s12943-021-01339-1, PMID: 33722265 PMC7957288

[88] Weide B Pascolo S Scheel B Derhovanessian E Pflugfelder A Eigentler TK . Direct injection of protamine-protected mRNA: results of a phase 1/2 vaccination trial in metastatic melanoma patients. J Immunother. (2009) 32:498–507. doi: 10.1097/CJI.0b013e3181a00068, PMID: 19609242

[89] Li RQ Wang W Yan L Song LY Guan X Zhang W . Identification of tumor antigens and immune subtypes in breast cancer for mRNA vaccine development. Front Oncol. (2022) 12:973712. doi: 10.3389/fonc.2022.973712, PMID: 36226063 PMC9548593

[90] Leko V Rosenberg SA . Identifying and targeting human tumor antigens for T cell-based immunotherapy of solid tumors. Cancer Cell. (2020) 38:454–72. doi: 10.1016/j.ccell.2020.07.013, PMID: 32822573 PMC7737225

[91] Li WH Li YM . Chemical strategies to boost cancer vaccines. Chem Rev. (2020) 120:11420–78. doi: 10.1021/acs.chemrev.9b00833, PMID: 32914967

[92] Lang F Schrörs B Löwer M Türeci Ö Sahin U . Identification of neoantigens for individualized therapeutic cancer vaccines. Nat Rev Drug Discov. (2022) 21:261–82. doi: 10.1038/s41573-021-00387-y, PMID: 35105974 PMC7612664

[93] Abelin JG Keskin DB Sarkizova S Hartigan CR Zhang W Sidney J . Mass spectrometry profiling of HLA-associated peptidomes in mono-allelic cells enables more accurate epitope prediction. Immunity. (2017) 46:315–26. doi: 10.1016/j.immuni.2017.02.007, PMID: 28228285 PMC5405381

[94] Alspach E Lussier DM Miceli AP Kizhvatov I DuPage M Luoma AM . MHC-II neoantigens shape tumour immunity and response to immunotherapy. Nature. (2019) 574:696–701. doi: 10.1038/s41586-019-1671-8, PMID: 31645760 PMC6858572

[95] Liu H Francis L Dagley LF Cobbold SA Webb AI Komander D . Major histocompatibility class II in murine antigen presenting cells is modified with a branched K63 and K11-linked ubiquitin chain. Sci Rep. (2025) 15:41884. doi: 10.1038/s41598-025-25817-4, PMID: 41290815 PMC12647164

[96] Ma C Balaban M Liu J Chen S Wilson MJ Sun CH . Inferring allele-specific copy number aberrations and tumor phylogeography from spatially resolved transcriptomics. Nat Methods. (2024) 21:2239–47. doi: 10.1038/s41592-024-02438-9, PMID: 39478176 PMC11621028

[97] Jurtz V Paul S Andreatta M Marcatili P Peters B Nielsen M . NetMHCpan-4.0: improved peptide-mhc class I interaction predictions integrating eluted ligand and peptide binding affinity data. J Immunol. (2017) 199:3360–8. doi: 10.4049/jimmunol.1700893, PMID: 28978689 PMC5679736

[98] Floudas CS Sarkizova S Ceccarelli M Zheng W . Leveraging mRNA technology for antigen based immuno-oncology therapies. J Immunother Cancer. (2025) 13:e010569. doi: 10.1136/jitc-2024-010569, PMID: 39848687 PMC11784169

[99] O’Donnell TJ Rubinsteyn A Bonsack M Riemer AB Laserson U Hammerbacher J . MHCflurry: open-source class I MHC binding affinity prediction. Cell Syst. (2018) 7:129–132.e4. doi: 10.1016/j.cels.2018.05.014, PMID: 29960884

[100] Zhao W Sher X . Systematically benchmarking peptide-MHC binding predictors: from synthetic to naturally processed epitopes. PLoS Comput Biol. (2018) 14:e1006457. doi: 10.1371/journal.pcbi.1006457, PMID: 30408041 PMC6224037

[101] Blaha DT Anderson SD Yoakum DM Hager MV Zha Y Gajewski TF . High-throughput stability screening of neoantigen/HLA complexes improves immunogenicity predictions. Cancer Immunol Res. (2019) 7:50–61. doi: 10.1158/2326-6066.CIR-18-0395, PMID: 30425106 PMC6324732

[102] Rasmussen M Fenoy E Harndahl M Kristensen AB Nielsen IK Nielsen M . Pan-specific prediction of peptide-MHC class I complex stability, a correlate of T cell immunogenicity. J Immunol. (2016) 197:1517–24. doi: 10.4049/jimmunol.1600582, PMID: 27402703 PMC4976001

[103] Huang X Zhang G Tang T Liang T . Identification of tumor antigens and immune subtypes of pancreatic adenocarcinoma for mRNA vaccine development. Mol Cancer. (2021) 20:44. doi: 10.1186/s12943-021-01310-0, PMID: 33648511 PMC7917175

[104] Weng Y Li C Yang T Hu B Zhang M Guo S . The challenge and prospect of mRNA therapeutics landscape. Biotechnol Adv. (2020) 40:107534. doi: 10.1016/j.bioteChadv.2020.107534, PMID: 32088327

[105] Linares-Fernández S Lacroix C Exposito JY Verrier B . Tailoring mRNA vaccine to balance innate/adaptive immune response. Trends Mol Med. (2020) 26:311–23. doi: 10.1016/j.molmed.2019.10.002, PMID: 31699497

[106] Diken M Kreiter S Selmi A Britten CM Huber C Türeci Ö . Selective uptake of naked vaccine RNA by dendritic cells is driven by macropinocytosis and abrogated upon DC maturation. Gene Ther. (2011) 18:702–8. doi: 10.1038/gt.2011.17, PMID: 21368901

[107] Wan X Shi W Ma L Wang L Zheng R He J . A 3′-pre-tRNA-derived small RNA tRF-1-Ser regulated by 25(OH)D promotes proliferation and stemness by inhibiting the function of MBNL1 in breast cancer. Clin Transl Med. (2024) 14:e1681. doi: 10.1002/ctm2.1681, PMID: 38725048 PMC11082093

[108] Victor MP Acharya D Begum T Ghosh TC . The optimization of mRNA expression level by its intrinsic properties—insights from codon usage pattern and structural stability of mRNA. Genomics. (2019) 111:1292–7. doi: 10.1016/j.ygeno.2018.08.009, PMID: 30179657

[109] Salleh MZ Norazmi MN Deris ZZ . Immunogenicity mechanism of mRNA vaccines and their limitations in promoting adaptive protection against SARS-CoV-2. PeerJ. (2022) 10:e13083. doi: 10.7717/peerj.13083, PMID: 35287350 PMC8917804

[110] Zhang H Zhang L Lin A Xu C Li Z Liu K . Algorithm for optimized mRNA design improves stability and immunogenicity. Nature. (2023) 621:396–403. doi: 10.1038/s41586-023-06127-z, PMID: 37130545 PMC10499610

[111] Reichmuth AM Oberli MA Jaklenec A Langer R Blankschtein D . mRNA vaccine delivery using lipid nanoparticles. Ther Deliv. (2016) 7:319–34. doi: 10.4155/tde-2016-0006c1, PMID: 27075952 PMC5439223

[112] Delaunay S Helm M Frye M . RNA modifications in physiology and disease: towards clinical applications. Nat Rev Genet. (2024) 25:104–22. doi: 10.1038/s41576-023-00645-2, PMID: 37714958

[113] Nance KD Meier JL . Modifications in an emergency: the role of N1-methylpseudouridine in COVID-19 vaccines. ACS Cent Sci. (2021) 7:748–56. doi: 10.1021/acscentsci.1c00197, PMID: 34075344 PMC8043204

[114] Oh A Lee S Park HJ Yoon S Ha D Lee J . Loop structure in poly(A) tail of mRNA vaccine enhances antigen translation efficiency and mRNA stability. NPJ Vaccines. (2025) 10:234. doi: 10.1038/s41541-025-01287-7, PMID: 41249784 PMC12623927

[115] Kim SC Sekhon SS Shin WR Ahn G Cho BK Ahn JY . Modifications of mRNA vaccine structural elements for improving mRNA stability and translation efficiency. Mol Cell Toxicol. (2022) 18:1–8. doi: 10.1007/s13273-021-00171-4, PMID: 34567201 PMC8450916

[116] Quan Y Yang H Li W Li L . mRNA vaccines: immunogenicity and quality characteristics. J Nanobiotechnol. (2025) 24:6. doi: 10.1186/s12951-025-03800-5, PMID: 41310620 PMC12765321

[117] Tarahovsky YS Arsenault AL MacDonald RC McIntosh TJ Epand RM . Electrostatic control of phospholipid polymorphism. Biophys J. (2000) 79:3193–200. doi: 10.1016/S0006-3495(00)76552-0, PMID: 11106623 PMC1301194

[118] Tarahovsky YS Koynova R MacDonald RC . DNA release from lipoplexes by anionic lipids: correlation with lipid mesomorphism, interfacial curvature, and membrane Fusion. Biophys J. (2004) 87:1054–64. doi: 10.1529/biophysj.104.042895, PMID: 15298910 PMC1304446

[119] Martinon F Krishnan S Lenzen G Magné R Gomard E Guillet JG . Induction of virus-specific cytotoxic T lymphocytes *in vivo* by liposome-entrapped mRNA. Eur J Immunol. (1993) 23:1719–22. doi: 10.1002/eji.1830230749, PMID: 8325342

[120] Luo C Sun J Sun B He Z . Prodrug-based nanoparticulate drug delivery strategies for cancer therapy. Trends Pharmacol Sci. (2014) 35:556–66. doi: 10.1016/j.tips.2014.09.008, PMID: 25441774

[121] Bonehill A Tuyaerts S Van Nuffel AM Heirman C Bos TJ Fostier K . Enhancing the T-cell stimulatory capacity of human dendritic cells by co-electroporation with CD40L, CD70 and constitutively active TLR4 encoding mRNA. Mol Ther. (2008) 16:1170–80. doi: 10.1038/mt.2008.77, PMID: 18431362

[122] Kong B Kim Y Kim EH Suk JS Yang Y . mRNA: A promising platform for cancer immunotherapy. Adv Drug Delivery Rev. (2023) 199:114993. doi: 10.1016/j.addr.2023.114993, PMID: 37414361 PMC11797636

[123] De Keersmaecker B Claerhout S Carrasco J Bar I Corthals J Wilgenhof S . TriMix and tumor antigen mRNA electroporated dendritic cell vaccination plus ipilimumab: link between T-cell activation and clinical responses in advanced melanoma. J Immunother Cancer. (2020) 8:e000329. doi: 10.1136/jitc-2019-000329, PMID: 32114500 PMC7057443

[124] Boudreau JE Bonehill A Thielemans K Wan Y . Engineering dendritic cells to enhance cancer immunotherapy. Mol Ther. (2011) 19:841–53. doi: 10.1038/mt.2011.57, PMID: 21468005 PMC3098642

