# Supplementary figures and images for: mRNA vaccines transform personalized lung cancer treatment
Source: Front Immunol. 2026 Feb 12;16:1707654. doi: 10.3389/fimmu.2025.1707654 (PMC12936860; doi:10.3389/fimmu.2025.1707654)

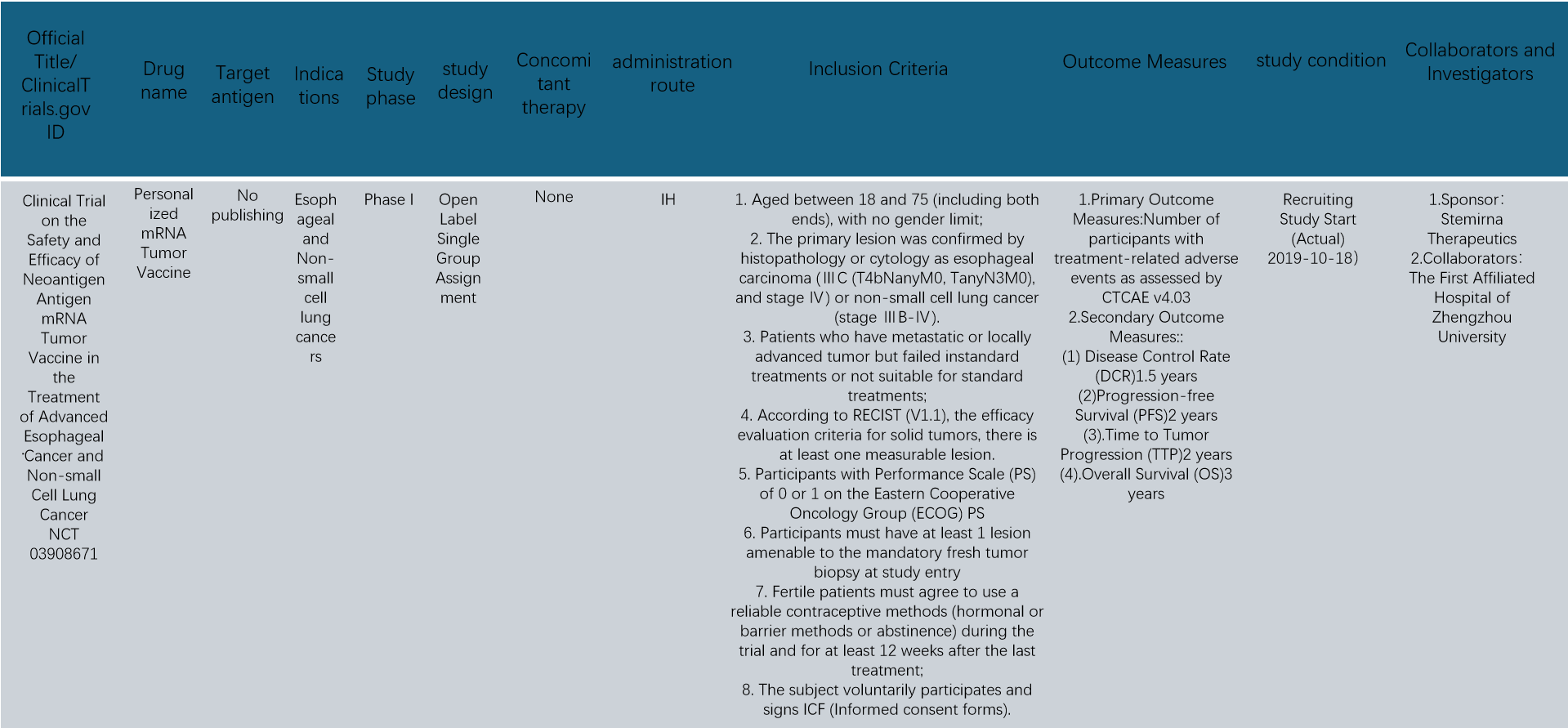

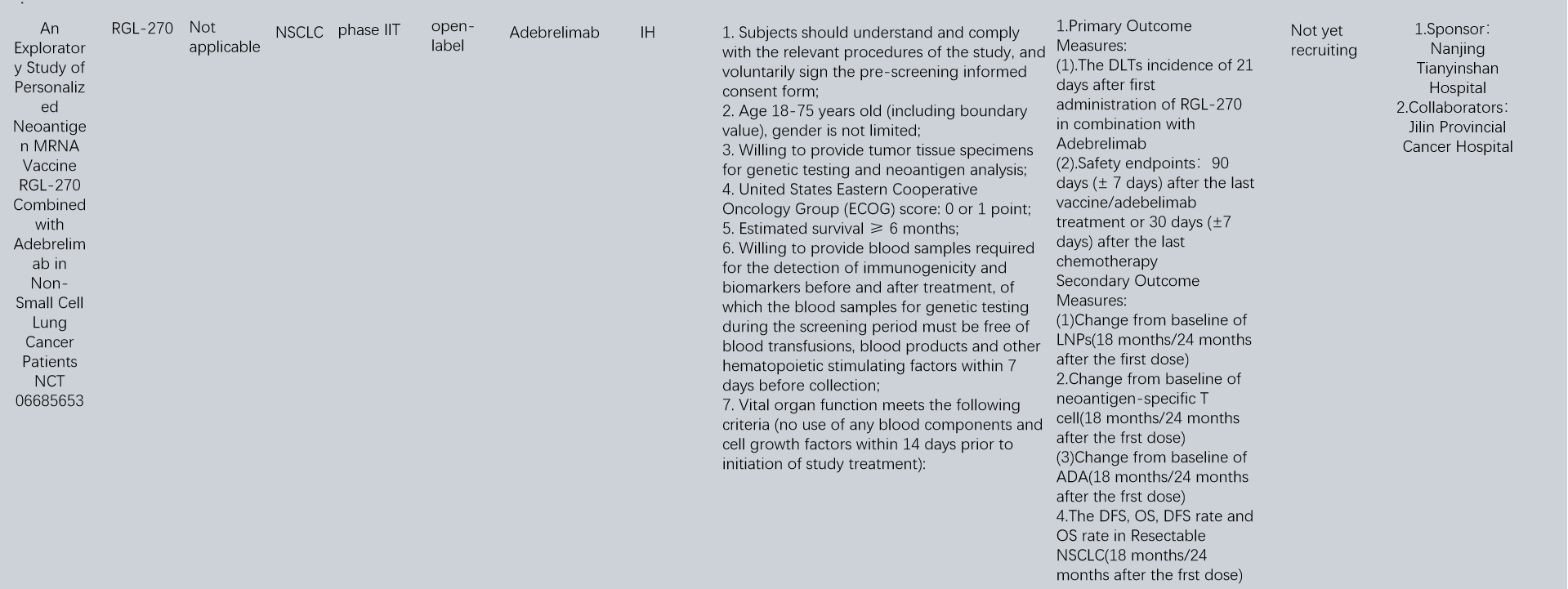

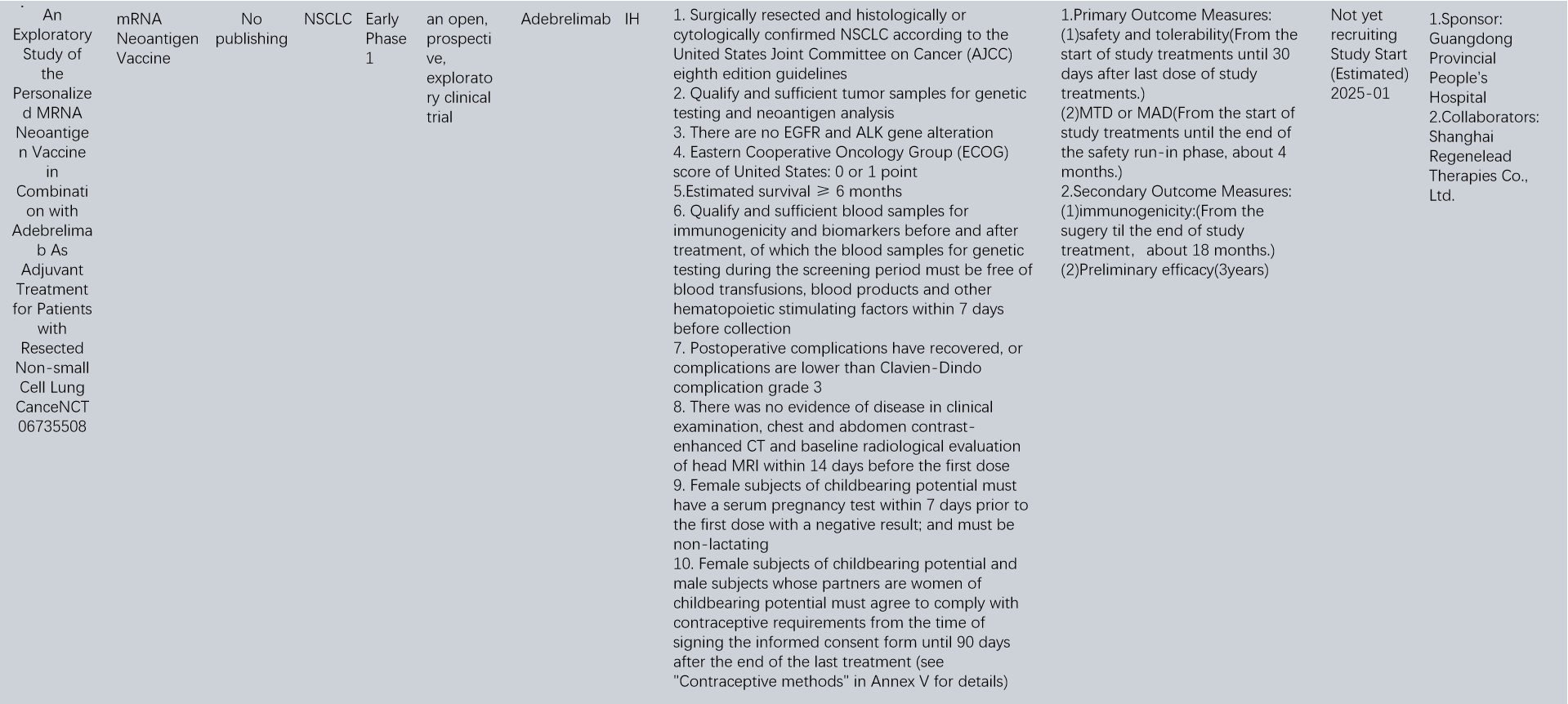

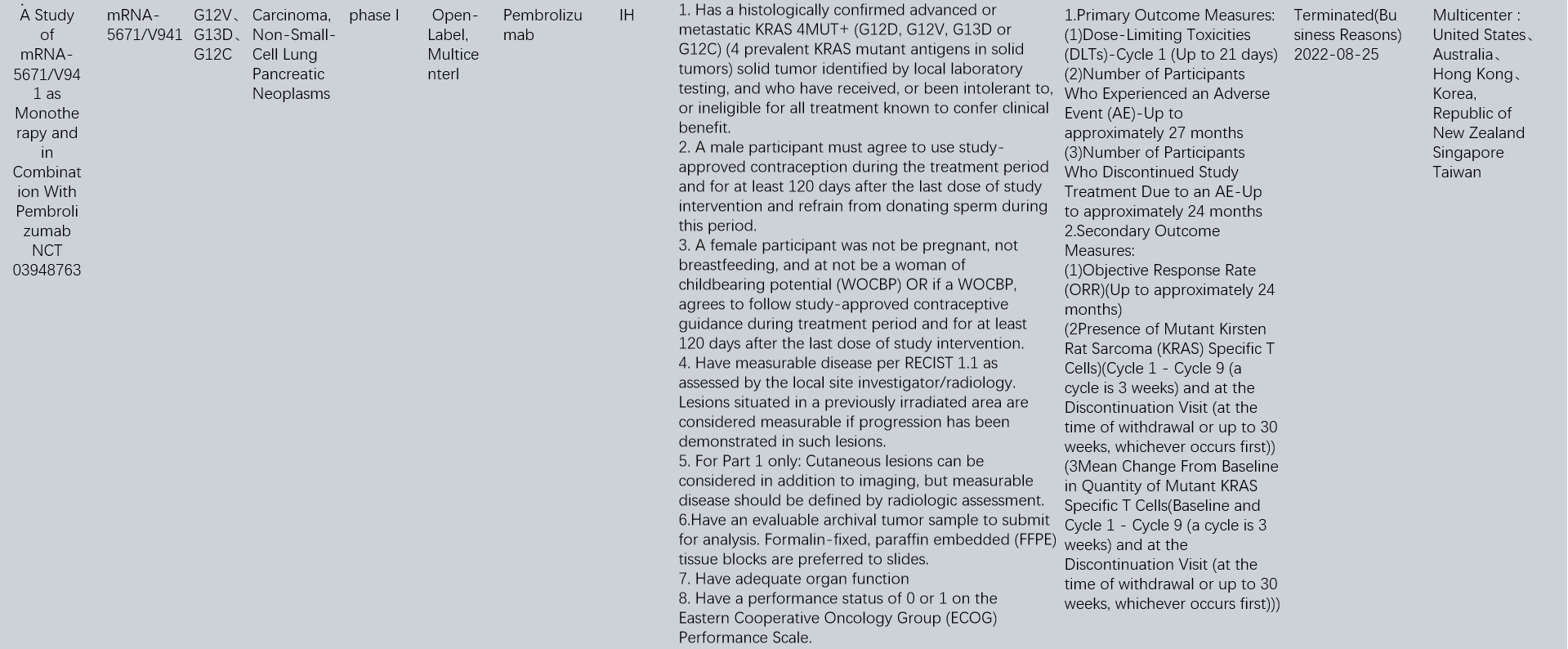

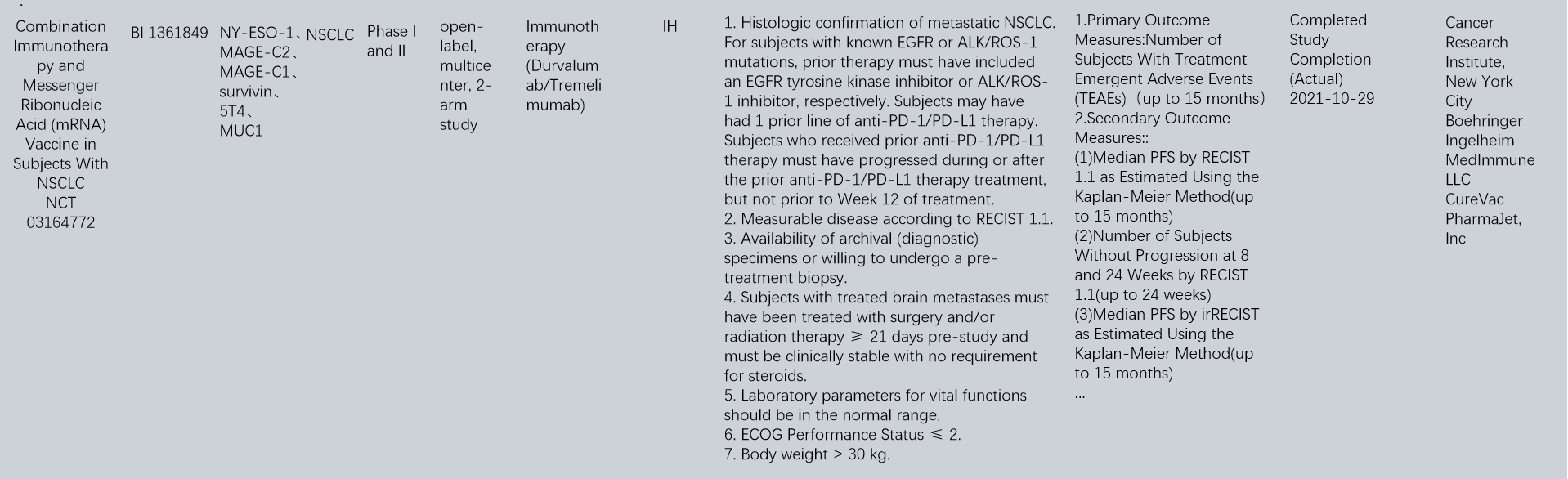


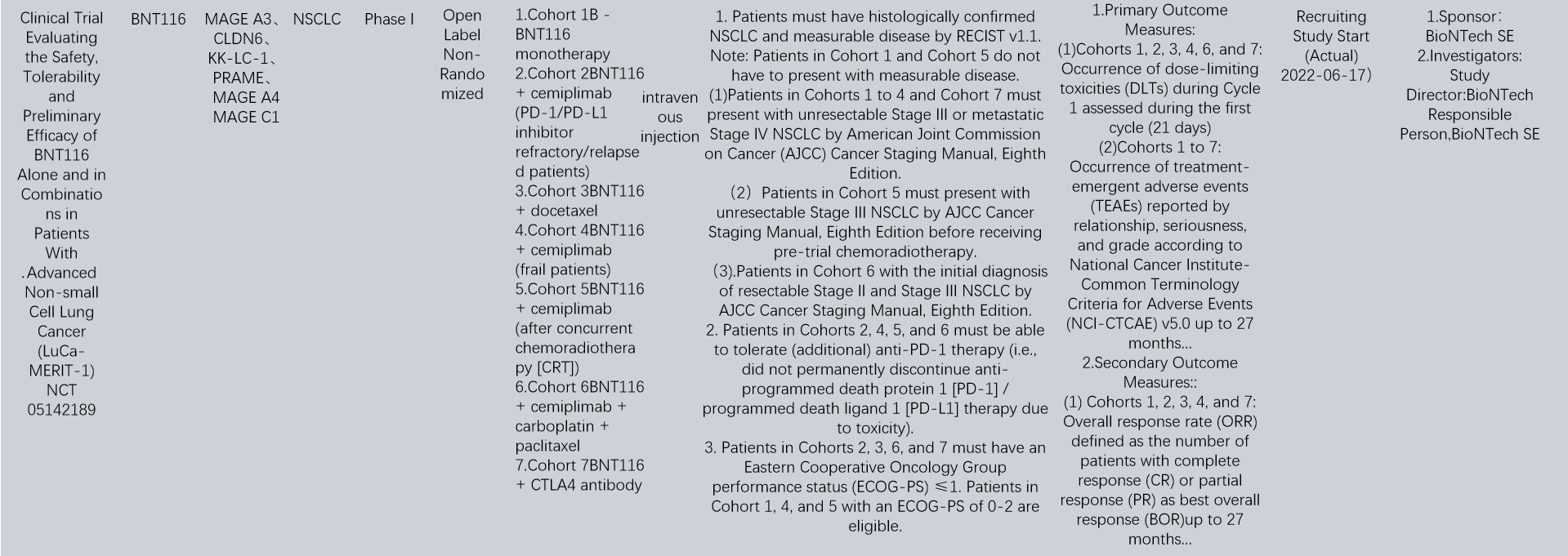


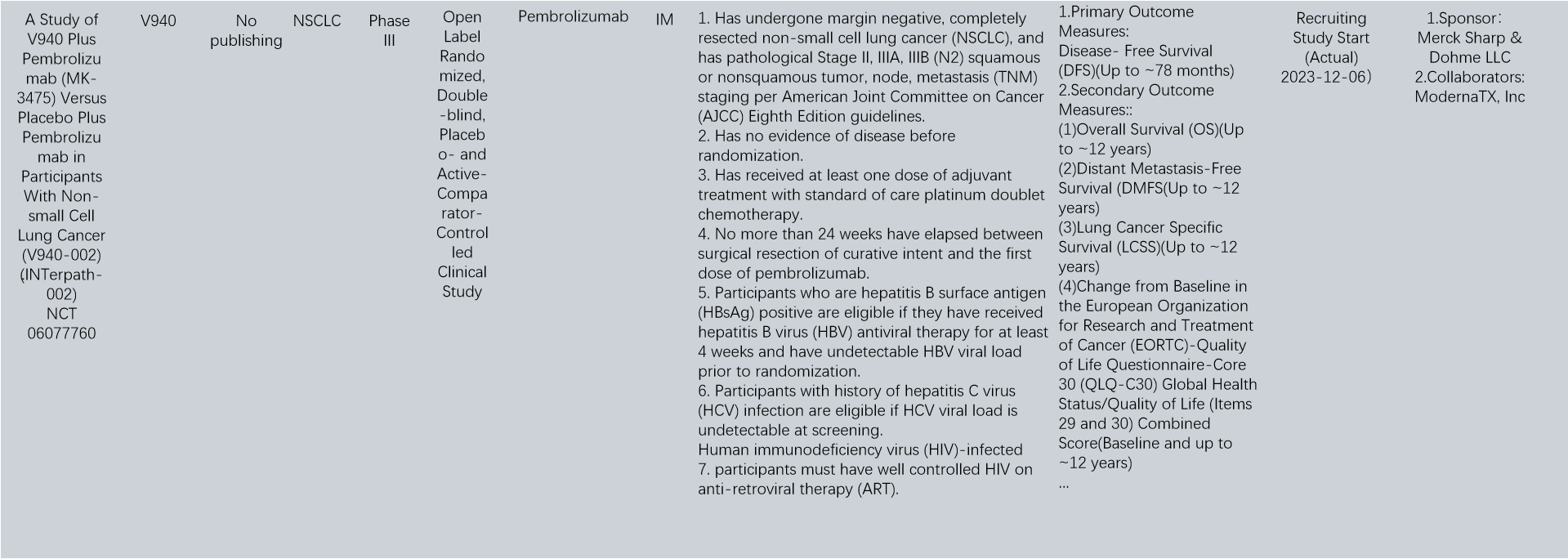

Supplement: Supplementary file 1 [file Table1.docx]
